# Drug Repurposing for Treating NCOR‐Related Intellectual Disability

**DOI:** 10.1002/advs.78030

**Published:** 2026-10-11

**Authors:** Yan Kong, Wenjun Zhou, Yuxiang Sun, Zihan Huang, Zhiyue Liu, Udhaya Kumar. S, Chenyan Li, Yi Lu, Chengchuang Song, Veronica C. Ciliberto, Makenna Mack, Lana E. Kim, Zhaoyong Hu, Fang Luo, Aihua Cao, Zheng Sun

**Affiliations:** ^1^ Department of Biochemistry and Molecular Biology School of Medicine Southeast University Nanjing Jiangsu China; ^2^ Department of Medicine – Division of Endocrinology Baylor College of Medicine Houston Texas USA; ^3^ Department of Pediatrics Shandong University Qilu Hospital Jinan Shandong China; ^4^ MyGenostics Inc. Beijing China; ^5^ Department of Molecular and Cellular Biology Baylor College of Medicine Houston Texas USA

**Keywords:** allopregnanolone, downregulation and upregulation, drug repositioning, haploinsufficiency, intellectual disability, knockout mouse, mecp2, neuregulin 1, neuroactive steroid, spironolactone

## Abstract

Nuclear receptor corepressors (NCOR1/2) mediate the transcriptional activity of methyl‐CpG binding protein 2 (MECP2), and genetic variants in *NCOR1/2* or *MECP2* associate with autism or intellectual disability. However, it is unknown whether heterozygous NCOR mutations impair cognitive functions and how NCOR‐related intellectual disability can be treated. Here, we constructed a knock‐in (KI) mouse line carrying a heterozygous *NCOR1* mutation (*NCOR1* c.2182+2T>G) found in a pediatric patient. These NCOR1^KI/+^ mice showed social avoidance, stereotypic behaviors, and memory deficits, similar to heterozygous *NCOR1* knockout mice, demonstrating *NCOR1* haploinsufficiency. snRNA‐seq revealed upregulated neuregulins (*Nrg1/3*) and downregulated γ‐aminobutyric acid (GABA) receptor subunit (*Gabra2*) in hypothalamic GABAergic neurons in NCOR1^KI/+^ mice. In cultured neurons, *Nrg1* overexpression is sufficient to downregulate *Gabra2*. Spironolactone, an NRG antagonist, prevents *Gabra2* downregulation induced by full‐length NRG1, but not by the NRG1 intracellular domain, suggesting that the noncanonical NRG backward signaling contributes to *Gabra2* downregulation upon *NCOR1* loss‐of‐function. Spironolactone and the GABA‐A receptor positive modulator allopregnanolone, both FDA‐approved drugs, mitigated cognitive impairments in NCOR1^KI/+^ mice. Furthermore, spironolactone improved learning in an autistic patient bearing a pathogenic mutation in *MECP2*. These findings elucidate the mechanism of NCOR‐related cognitive dysfunctions and provide preclinical proof‐of‐concept for treatment through drug repurposing.

## Graphical abstract

An autism‐associated NCOR1 heterozygous mutation upregulates NRGs and activates NRG backward signaling in hypothalamic GABAergic neurons in mouse models, leading to Gabra2 downregulation and cognitive deficits, which can be therapeutically targeted by repurposing FDA‐approved drugs.

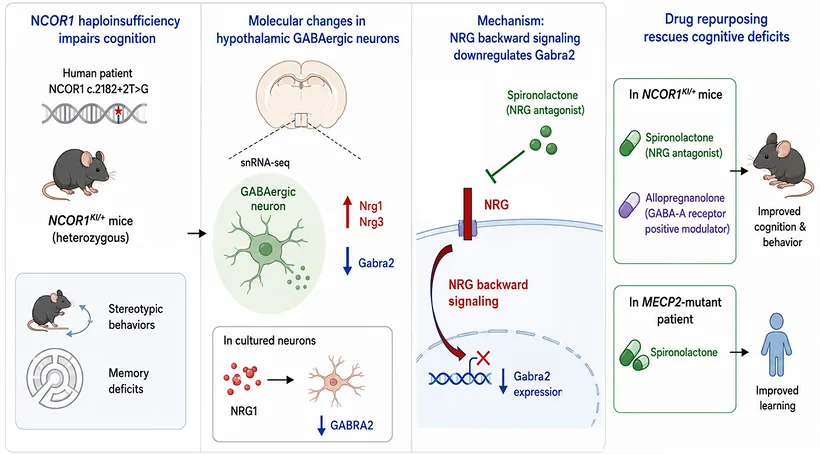

## 1 Introduction

Autism spectrum disorder (ASD) is a neurodevelopmental disorder characterized by a complex interplay of genetic and environmental factors. Approximately 30% of individuals with ASD have intellectual disability [1, 2, 3]. Rett syndrome (RTT) is the most debilitating form of ASD, caused by mutations [4, 5] or copy number changes [6, 7, 8] in the methyl‐CpG‐binding protein 2 (*MECP2*) gene, which plays essential roles in maintaining mature neuronal networks and proper brain functions [6, 9, 10, 11]. MECP2 forms a protein complex with nuclear receptor corepressors (NCOR1/2) and histone deacetylase 3 (HDAC3), which alters epigenetic modifications, remodels chromatin architecture, and regulates gene transcription [12]. Disrupting the protein interaction between MECP2 and NCORs is postulated to underlie the pathogenesis of RTT [13, 14].

The mechanisms by which NCORs regulate cognitive functions are not yet fully understood. Our previous study showed that whole‐body knock‐in of *NCOR1/2* deacetylase‐activating domain point mutations (NS‐DADm) in mice abolished HDAC3 deacetylase activity due to the disruption of the NCOR1/2‐HDAC3 interaction [15, 16], leading to impairments in multiple cognitive tasks accompanied by the down‐regulation of γ‐aminobutyric acid type A receptor subunit α2 (*Gabra2*) in the lateral hypothalamus (LH). Genetic deletion of *Ncor1/2*, specifically in GABAergic neurons with *Vgat*‐Cre (NS‐V mice), recapitulated the cognitive impairments observed in the NS‐DADm mouse model. Notably, GABRA2 subunit expression was also downregulated in GABAergic neurons of the LH from NS‐V mice. This was associated with hyperactive LH^GABA^ neurons, which impairs hippocampal long‐term potentiation (LTP) and memory through a LH^GABA^‐to‐hippocampal CA3 monosynaptic projection [15]. Patients carrying pathogenic mutations of *NCOR1/2* show both intellectual disability and developmental retardation [17], suggesting the critical role of NCOR1/2 in regulating cognitive functions in humans.

However, although patients carry heterozygous mutations in a single gene (NCOR1 or NCOR2), our previous mouse models are based on homozygous depletion or site mutations of both NCOR1 and NCOR2. Therefore, several key questions remain unanswered. Is NCOR1 or NCOR2 haploinsufficient in maintaining cognitive function? Are the mutations in a single gene of NCOR1 identified in human patients sufficient to cause intellectual disability? Since NCOR1/2 are general transcriptional corepressors, how does NCOR loss‐of‐function downregulate GABA‐A receptor gene expression? Could NCOR mutation‐related cognitive dysfunction be treated through drug repurposing? Here, we constructed two new genetically modified mouse lines of NCOR1 and integrated single‐nucleus transcriptomics, pharmacologic intervention, and in vitro cell culture models to address these questions. Furthermore, we tested drug repurposing in an autistic patient bearing pathogenic mutations in MECP2, the critical component of the NCOR complex.

## 2 Results

### 2.1 Haploinsufficiency of *Ncor1* Leads to Cognitive Impairments and Repetitive Behaviors

Our previous NCOR mutation mouse models, including NS‐DADm and NS‐V mice, were developed by homozygous manipulations of both *Ncor1* and *Ncor2* genes. However, most disease‐associated *NCOR1/2* mutations in ASD patients were identified as heterozygous in only a single gene. Therefore, we first addressed whether *NCOR1* was haploinsufficient for cognitive functions. The focus on NCOR1 is based on the patient's availability. We do not have evidence suggesting that NCOR1 is more dominant than NCOR2. We crossbred *Ncor1 ^loxP /+^* mice with β‐actin‐*Cre* transgenic mice, resulting in heterozygous *Ncor1* whole‐body knockout (NCOR1^+/−^) mice (Figure 1A). The Cre allele was then bred out of the colony. The NCOR1^+/−^ mice were born at the Mendelian ratio and had body weights similar to those of their wild‐type (WT) littermates (Figure 1B). NCOR1^+/−^ mice displayed impaired performance in the novel object recognition (NOR) test (Figure 1C–E), characterized by reduced exploration time on the novel object compared to the familiar object (Figure 1C), without affecting overall exploration behavior compared to WT littermates (Figure 1D). NCOR1^±^ mice also exhibited impaired social memory in the 3‐chamber social memory (3CH‐SM) test (Figure 1F–H). In contrast, baseline social interaction in the 3‐chamber social interaction (3CH‐SI) test was largely preserved (Figure 1I), despite a mild reduction in exploratory behavior in this test (Figure 1J). In the Morris water maze (MWM) test (Figure 1K–M), NCOR1^+/−^ mice took a longer time to find the rescue platform during learning trials (Figure 1K) and spent less time in the target zone where the rescue platform was located (Figure 1L), indicating impaired ability in spatial learning and memory. Additionally, NCOR1^+/−^ mice displayed lower anxiety levels in the elevated plus maze (EPM) test (Figure 1N–P), as evidenced by increased time spent in the open arms of the EPM, but did not show abnormality in the open field arena (OFA) test (Figure 1Q,R). Interestingly, NCOR1^+/−^ mice exhibited stereotypic behaviors characterized by increased marble‐burying behaviors (Figure 1S,T). These findings suggest that NCOR1 haploinsufficiency leads to cognitive dysfunctions and repetitive behaviors.

**FIGURE 1:**
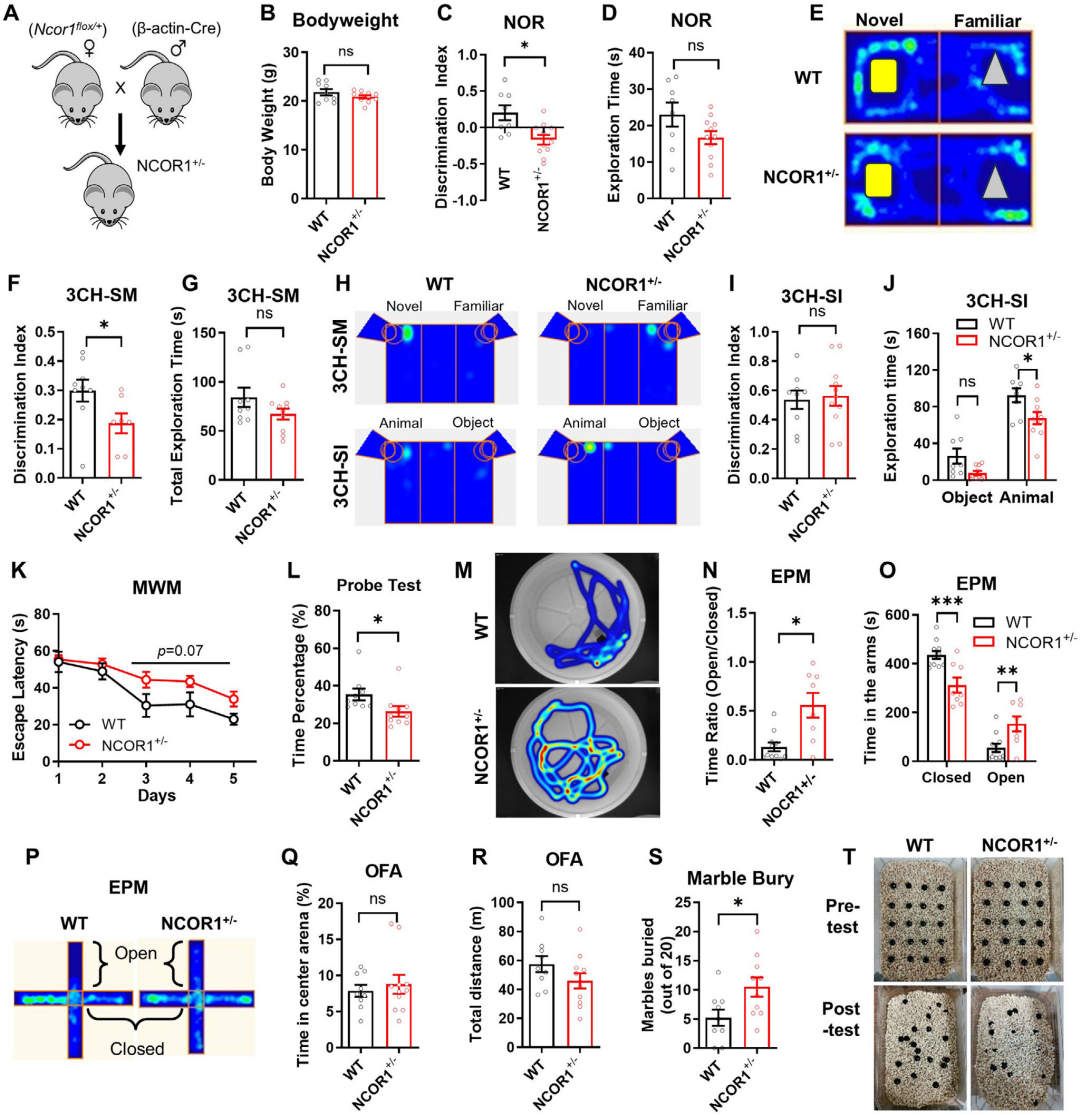
Haploinsufficiency of *Ncor1* leads to cognitive impairments and repetitive behaviors. (A) Diagram of generating NCOR1^+/−^ mouse. (B) Body weight of female WT and NCOR1^+/−^ mice at 3–4 months old. (C‐E) Discrimination index, total exploration time, and trace of exploration activity in the novel object recognition (NOR) test. (F–H) Discrimination index, total exploration time, and trace of exploration activity in the 3‐chamber social memory (3CH‐SM) test. (I, J) Social preference index and total exploration time in the 3‐chamber social interaction (3CH‐SI) test. (K) Escape latency of the Morris water maze (MWM) test during the 5‐day training. Two‐way repeated ANOVA was used. (L, M) Time percentage spent in the target quadrant zone and a heatmap during the MWM probe test. (N–P) The ratio of time in open arms to time in closed arms, total time spent in either arm, and trace of exploration activity in the elevated plus maze (EPM) test. (Q, R) Time spent in the central area and total travel distance during the open field arena (OFA) test. (S, T) Number of buried marbles and representative picture in the marble bury (MB) test. The sample size is indicated by individual data points (n = 6–11 per group). Each data point is one mouse. Data are mean ± SEM. Data were analyzed by a two‐tailed t‐test, 2‐way ANOVA, and 1‐way repeated ANOVA, unless otherwise indicated. ^*^ *p* < 0.05, ^**^ *p* < 0.01, and ^***^ *p* < 0.001.

### 2.2 Heterozygous Knock‐in Mice Carrying an ASD‐Associated *NCOR1* Variant Display Cognitive Dysfunctions

The patient harboring the heterozygous c.2182+2T>G variant in *NCOR1* exhibits global developmental delay, moderate learning difficulties, and features of ASD [15, 18]. Another patient with ASD also carries a *de novo* heterozygous mutation at the adjacent splice donor site of *NCOR1* (c.2182+1G>T) [19]. These mutations are predicted to cause exon skipping and a reading‐frame shift, leading to a truncated NCOR1 protein or reduced protein production. The truncated protein of 727 amino acids is predicted to lose the repression domains 2 and 3 (RD2/3) and all receptor interaction domains on the C‐terminus of NCOR1. Using the embryonic stem cell‐based gene targeting strategy, we engineered a novel mouse heterozygous knock‐in (KI) model carrying the analogous *NCOR1* c.2182+2T>G mutation (KI/+) (Figure 2A). The genotype of NCOR1^KI/+^ mice was confirmed by PCR of genomic DNA followed by Sanger sequencing of the 725‐bp band flanking the variant site (Figure 2B). The NCOR1^KI/+^ mice were born at the Mendelian ratio. The protein level of NCOR1 in the brain was reduced in NCOR1^KI/+^ mice compared to WT littermate (Figure 2C,D). We did not detect the truncated form of the NCOR1 protein. The mRNA level of *Ncor1* was also lower than that of the WT control (Figure 2E), suggesting that the mutation likely triggers nonsense‐mediated mRNA decay or protein degradation. Male NCOR1^KI/+^ mice displayed slightly reduced body weight compared to the WT littermates (Figure 2F) but retained normal motor coordination and locomotor activity, as characterized by similar retention time in the rotor‐rod test (Figure 2G), muscle grip strength (Figure 2H), and behaviors in the OFA test (Figure 2I,J). However, NCOR1^KI/+^ mice showed impaired memory in the NOR test (Figure 2K,L) and the object‐location recognition (OLR) test (Figure 2M,N). NCOR1^KI/+^ mice also showed impaired social memory in the 3CH‐SM test (Figure 2O,P) without affecting the baseline social interaction in the 3CH‐SI test (Figure 2Q,R). NCOR1^KI/+^ mice also showed impaired learning and memory in the MWM test (Figure 2S,T). The exploratory behaviors of NCOR1^KI/+^ mice remained normal in all these tests (Figure 2J,L,N,P,R). NCOR1^KI/+^ mice also showed normal anxiety levels in the EPM test (Figure 2U,V) and light‐dark (LD) test (Figure 2W,X). Additionally, NCOR1^KI/+^ mice exhibited increased stereotypic behaviors in the marble‐bury test (Figure 2Y). The overall similarity in behavioral phenotypes between NCOR1^±^ and NCOR1^KI/+^ mouse lines suggests that the ASD‐associated NCOR1 mutation causes cognitive impairment and stereotypic behaviors through heterozygous NCOR1 loss‐of‐function.

**FIGURE 2:**
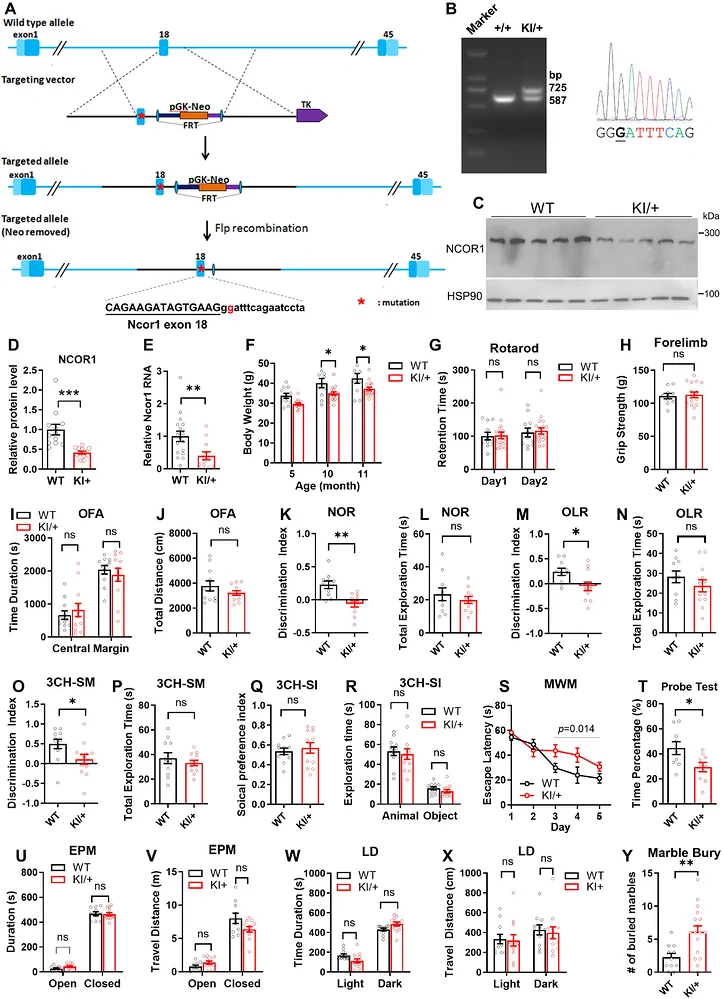
Heterozygous knock‐in mice carrying an ASD‐associated *NCOR1* variant display cognitive dysfunctions. (A) Targeting strategy. (B) Sanger sequencing validation of PCR products from F1 mouse #5. The c.2182+2T>G mutation site was underlined. (C, D) Western blot and quantification of NCOR1 protein level in the brain of WT and NCOR1^KI/+^ mice. (E) RT‐qPCR results displayed the downregulation of NCOR1 in the NCOR1^KI/+^ brain. (F) Body weight of male NCOR1^KI/+^ and WT mice. (G) Retention time in the rotarod (RR) test. (H) Grip strength of the forelimb. (I, J) Total travel distance and time spent in both central and marginal areas during OFA. Data were analyzed by a two‐tailed unpaired t‐test. (K, L) Discrimination index and total exploration time in the NOR test. Data were analyzed by a two‐tailed unpaired t‐test. (M, N) Discrimination index and total exploration time in the object location recognition (OLR) test. (O, P) Discrimination index and total exploration time in the 3CH‐SM test. (Q, R) Social preference index and total exploration time in the 3CH‐SI test. (S) Escape latency in the MWM test. Two‐way repeated ANOVA was used. (T) Time percentage spent in each quadrant during the probe test. (U, V) Time spent in the open or closed arms and total travel distance in the arms during the EPM test. (W, X) Time spent in the light or dark chambers and total travel distance during the light‐dark (LD) test. (Y) Number of buried marbles in the MB test. The sample size is indicated by the individual data points (n = 9–18 per group). Each data point is one mouse. Male mice at 3–6 months old were used. Data are mean ± SEM. Data were analyzed by a two‐tailed unpaired t‐test, 2‐way ANOVA, and 1‐way repeated ANOVA unless otherwise indicated. ^*^ *p* < 0.05, ^**^ *p* < 0.01, and ^***^ *p* < 0.001.

### 2.3 NCOR1 Deficiency Upregulates Neuregulin (*Nrg*) and Downregulates GABA‐A Signaling

To investigate the molecular mechanism underlying ASD‐like behaviors due to the human *NCOR1* mutation, we performed single‐nuclei RNA sequencing analysis (snRNA‐seq) of the hypothalamus from the heterozygous NCOR1^KI/+^ (nKI) mice and the NCOR1^+/+^ littermates (WT) at the age of 6 months (Figure 3A). After quality control and batch‐effect correction, we obtained 20,093 nuclei. Based on summary statistics from a genome‐wide association study (GWAS, EFO0003758) [20, 21], we found stronger enrichment of the autism trait in NCOR1^KI/+^ mice than in WT mice using Gene‐Set Variant Analysis (GSVA) (Figure 3B). Based on the canonical cell‐type markers (Figure S1A), we identified 12 distinct cell types, including neurons, astrocytes, oligodendrocytes, and microglia. The Uniform Manifold Approximation and Projection (UMAP) plots revealed differences in the neuron cluster between the WT and NCOR1^KI/+^ groups (Figure 3C). Consistently, among a total of 2,605 differentially expressed genes (DEGs) between NCOR1^KI/+^ and WT mice (Figure S1B), the top DEGs were primarily contributed by neurons (Figure 3D). Several of the top DEGs were related to neuronal cell fate, signal transduction, and nervous system development, including *Nrg1*, *Unc5d*, *Rasgrf2*, *Meis2*, *Syt6*, and *Htr4* [22, 23, 24, 25].

**FIGURE 3:**
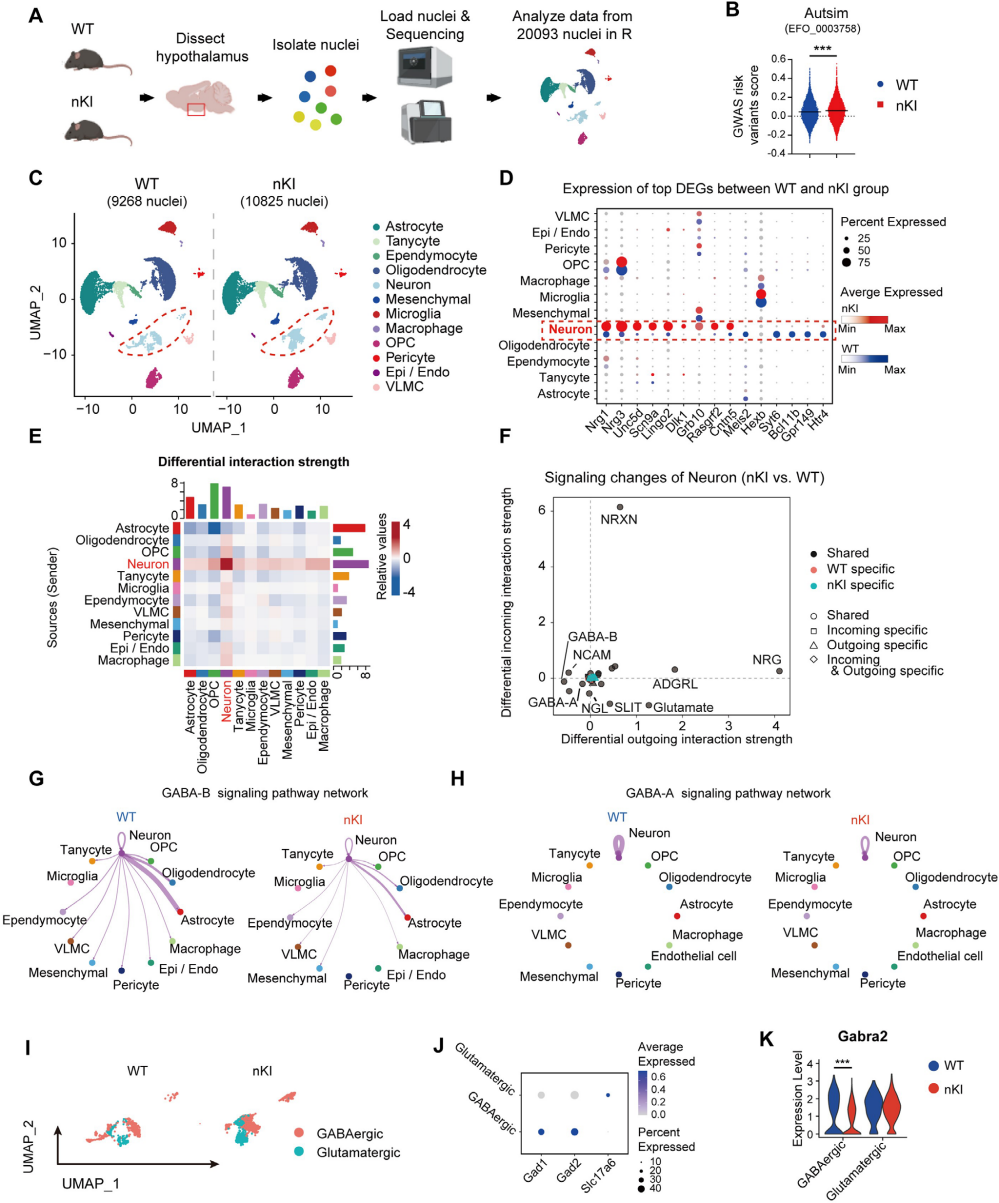
NCOR1 deficiency upregulates neuregulin (NRG) and downregulates GABA‐A signaling. (A) Pipeline of snRNA‐seq analysis of the hypothalamus from NCOR1^KI/+^ (nKI) and WT female mice at 6 months old. (B) GWAS risk variant score analysis of snRNA‐seq data for autism. (C) UMAP plot revealing 12 cell types. OPC, oligodendrocyte precursor; Epi/Endo, epithelial and endothelial cells; VLMC, vascular and leptomeningeal cells. (D) Dot plots show the top differentially expressed genes (DEGs) in NCOR1^KI/+^ vs. WT mice, mostly contributed by neurons. (E) CellChat results in communication strength between different cell types in NCOR1^KI/+^ vs. WT mice. (F) Scatter plot of different neuron interactions in NCOR1^KI/+^ vs. WT mice. (G, H) GABA‐B and GABA‐A signaling pathway networks in NCOR1^KI/+^ vs. WT mice. (I, J) GABAergic and glutamatergic neuron sub‐clusters identified in WT and NCOR1^KI/+^ mice. (K) *Gabra2* expression in GABA and glutamatergic neurons. Data were analyzed by a two‐tailed unpaired t‐test. ^***^ *p* < 0.001.

Given that communication among neurons, or between neurons and other cellular types, plays an important role in the functioning of the nervous system, we applied the CellChat algorithm [26] to explore differences in cell‐cell communication between WT and NCOR1^KI/+^ groups. The analysis revealed that neuron‐neuron communication was stronger in NCOR1^KI/+^ mice than in WT, whereas communication among other cell types was weaker in NCOR1^KI/+^ than in WT hypothalamus (Figure 3E). From the perspective of neurons, neuregulin (NRG) signaling emerged as the top upregulated outgoing pathway in NCOR1^KI/+^ compared to WT, while neurexin (NRXN) signaling is the top upregulated incoming pathway (Figure 3F). NRXN and neuroligins (NLGN) are cell‐adhesion molecules that connect pre‐ and postsynaptic neurons at synapses [27]. Dysfunction of the NRXN‐NLGN pathway influences neuronal activity in ASD pathogenesis [28]. In human induced pluripotent stem cell (iPSC)‐derived cortical neurons, ASD‐associated NRXN1 deletions selectively enhance excitatory synaptic signaling without affecting inhibitory synapses [29]. We did not focus on NRXN signaling because it appeared as an incoming pathway in neurons, not an outgoing one. Therefore, we reasoned that it probably reflects changes outside the hypothalamus region. Although beyond the scope of this study, the NCOR1–NRXN signaling axis represents a promising avenue for future investigation in ASD.

Among neurotransmitter‐mediated interactions, glutamate‐mediated communication was upregulated, while GABA‐mediated communication, including GABA‐A and GABA‐B receptor signaling, was downregulated in NCOR1^KI/+^ compared to WT mice (Figure 3F). We then performed CellChat analysis to infer ligand‐receptor interactions or cell‐cell communications. In the resultant circle plot (Figure 3G), nodes represent different cell types, distinguished by different colors, while lines between nodes indicate communication between cell types. Thicker lines suggest a higher interaction weight or probability. The line color is the same as the cell type expressing the ligand, which indicates the direction of the communication. The decrease of the GABA‐B signaling in NCOR1^KI/+^ vs. WT primarily results from neuron‐oligodendrocyte or neuron‐astrocyte crosstalk rather than neuron‐neuron communication (Figure 3G and Figure S1C). In contrast, changes in GABA‐A signaling mainly occurred in neuron‐neuron communication (Figure 3H and Figure S1D).

Interrogating the DEG analysis results, we found that GABA type A receptor subunit α2 (*Gabra2*) was decreased in neurons of NCOR1^KI+^ mice compared to WT mice (Figure S1E). Upon further sub‐clustering the neurons into GABAergic and glutamatergic neurons (Figure 3I,J), we observed that *Gabra2* downregulation (NCOR1^KI/+^ vs. WT) predominantly occurred in GABAergic neurons, but not in glutamatergic neurons (Figure 3K). To assess the effects of transcriptomic changes on neuronal firing, we performed immunostaining for cFos in brain sections. GABAergic neurons were identified by GAD67 co‐staining. Among GAD67^+^ neurons in the lateral hypothalamus (LH), KI/+ mice exhibit a higher percentage of cFos^+^ neurons than WT mice, suggesting increased neuronal activity of LH GABAergic neurons in KI/+ mice compared to WT mice (Figure S1F,G), which is in line with the downregulated GABA‐A receptor in these neurons. In summary, snRNA‐seq revealed that hypothalamic GABA neurons in NCOR1^KI/+^ mice exhibit upregulation of NRG signaling and downregulation of GABA‐A signaling, associated with increased spontaneous firing activity.

### 2.4 NRG Backward Signaling Contributes to NCOR1‐Mediated Regulation of the GABA‐A Receptor

As a nuclear receptor corepressor, NCOR1 is believed to mediate the repressive action of unliganded nuclear receptors and other transcription factors. However, NCOR1 loss‐of‐function downregulated the expression of GABA‐A receptors, such as *Gabra2*, as revealed by snRNA‐seq. Therefore, NCOR1 might indirectly regulate GABA receptor expression. We focused on NRG signaling, which emerged as the most upregulated outgoing pathway in neurons from NCOR1^KI/+^ mice compared to WT littermates (Figures 3F and 4A). Notably, DEG analysis from snRNA‐seq revealed that *Nrg1/3* was upregulated in GABAergic neurons in the hypothalamus of NCOR1^KI/+^ mice compared to WT (Figure 4B,C and Figure S1B). *Nrg1* and its receptors, ErbB receptor tyrosine kinases, are expressed in GABAergic neurons of the hypothalamus [30, 31] and are implicated in many mental disorders [32, 33, 34].

**FIGURE 4:**
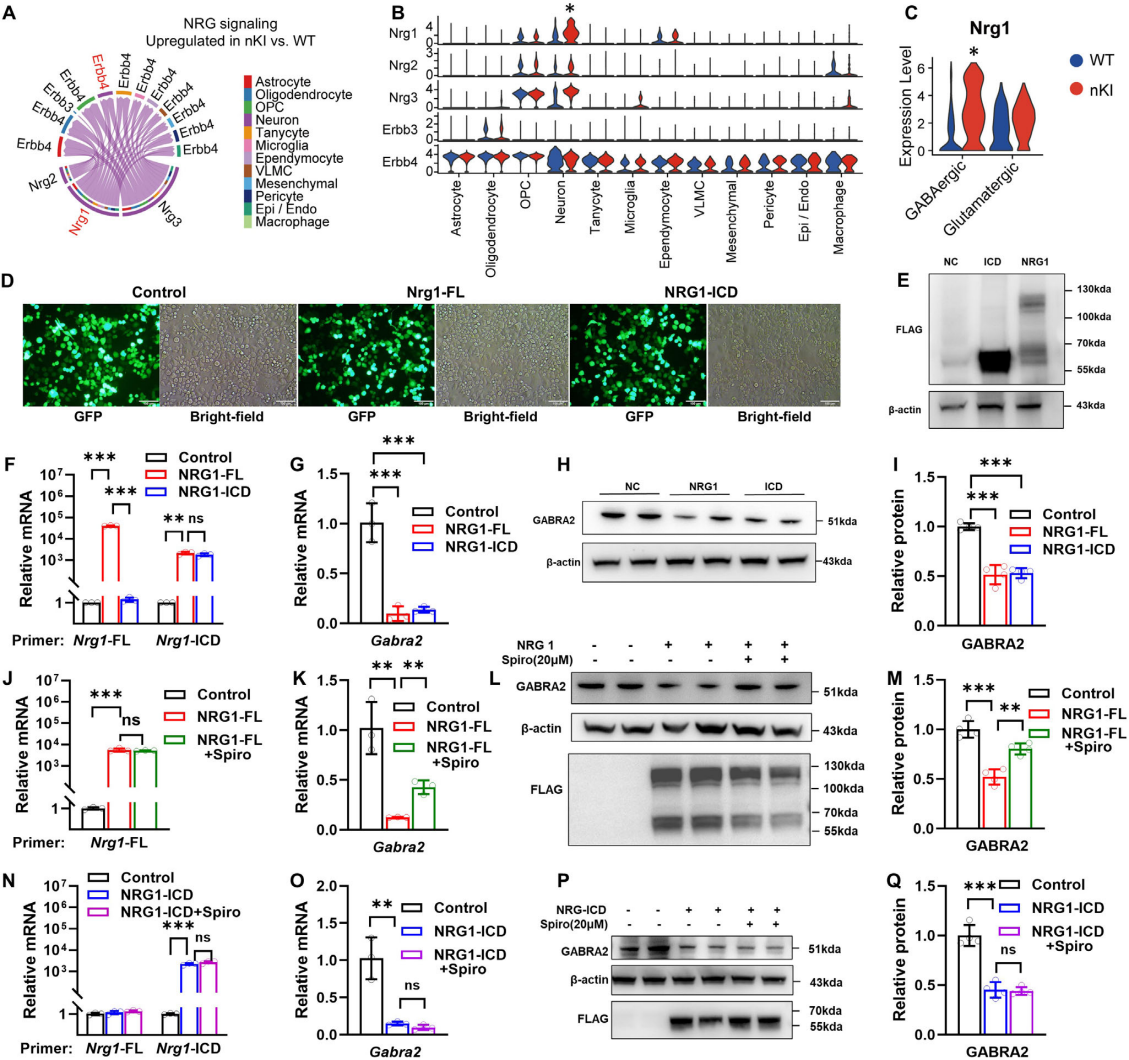
NRG backward signaling contributes to NCOR1‐mediated regulation of the GABA‐A receptor. (A) Cell‐cell communication analysis showing upregulated NRG signaling in the NCOR1^KI/+^ (nKI) vs. WT mice. (B) DEG analysis of upregulated gene expression of *Nrg1/3* in the NCOR1^KI/+^ vs. WT mice. (C) *Nrg1* expression in the GABAergic and glutamatergic neurons. ^*^ *p* <0.05 by a two‐tailed t‐test. (D) Representative images of Neuro‐2a (N2a) cells transfected with empty vector (control), full‐length Nrg1 (NRG1‐FL), or NRG1‐ICD plasmids. (E) Western blot of transfected N2a cells with anti‐Flag antibody. β‐actin was the loading control. (F) RT‐qPCR analysis of transfected N2a cells with NRG1‐FL primers (left) targeting the non‐ICD region or ICD‐specific primers (right). (G) RT‐qPCR analysis of *Gabra2* mRNA of transfected N2a cells. (H, I) Western blot and quantification of GABRA2 protein in N2a cells. (J, K) RT‐qPCR analysis of *Nrg1* and *Gabra2* mRNA after spironolactone (Spiro, 20 µM) treatment in N2a cells transfected with NRG1‐FL. (L, M) Western blot of GABRA2 protein in Spiro‐treated N2a cells transfected with NRG1‐FL. (N, O) RT‐qPCR analysis of *Nrg1* and *Gabra2* mRNA in Spiro‐treated N2a cells transfected with NRG1‐ICD. (P, Q) Western blot of GABRA2 protein in Spiro‐treated N2a cells transfected with NRG1‐ICD. n = 3 wells of cells for RT‐qPCR. n = 4 wells of cells for Western blot analysis. One‐way ANOVA and Tukey's post hoc test were used unless otherwise indicated. Data are mean ± SEM. ^*^ *p* < 0.05, ^**^ *p* < 0.01, and ^***^ *p* < 0.001.

NRG comprises a family of widely expressed epidermal growth factor (EGF)‐like proteins that can regulate gene transcription through both forward and backward signaling pathways. Canonical forward signaling refers to the extracellular domain of NRG binding to receptor tyrosine kinases of the ErbB family, which induces ErbB dimerization and downstream kinase activation. Noncanonical backward signaling refers to the NRG intracellular domain (NRG‐ICD) [22, 35], generated by γ‐secretase cleavage of the NRG C‐terminal fragment, which translocates into the nucleus to regulate gene expression. It has been reported that NRG1 reduced the expression of GABA‐A receptor α subunits in rat hippocampal slices [36], but the underlying mechanism is unclear.

To investigate the role of NRG in regulating GABRA2 expression, we conducted in vitro studies using Neuro‐2a cells, a mouse neural cell line. Cells were transfected with plasmids overexpressing Flag‐tagged full‐length mouse NRG1 (NRG1‐FL) or NRG1‐ICD. The NRG1‐FL primers target regions outside the ICD domain and could specifically amplify full‐length mouse *Nrg1* mRNA. In contrast, NRG1‐ICD primers detect the overexpression of both NRG1‐FL and NRG1‐ICD mRNA. After confirming that NRG1‐FL and NRG1‐ICD can be efficiently transfected and overexpressed (Figure 4D–F), we assessed the effects on *Gabra2* expression using both RT‐qPCR and western blot. *Gabra2* expression was downregulated at both the mRNA (Figure 4G) and protein levels (Figure 4H,I) following NRG1‐FL or NRG1‐ICD overexpression, suggesting that NRG signaling in neuronal cells suppresses *Gabra2* gene expression via an ICD‐mediated mechanism. Interestingly, co‐treatment with spironolactone (Spiro), an antagonist of NRG1‐ErbB interaction [37] (Figure 4J), partially blocked the NRG1‐FL‐mediated downregulation of *Gabra2* at the mRNA (Figure 4K) and protein level (Figure 4L,M). In contrast, spironolactone did not influence the NRG1‐ICD‐mediated *Gabra2* downregulation at the mRNA level (Figure 4N,O) or protein level (Figure 4P,Q). These results suggest that NCOR1 loss‐of‐function leads to upregulation of *Nrg1* expression, which subsequently downregulates *Gabra2* expression through the noncanonical NRG backward signaling pathway.

To explore the transcriptional regulatory mechanisms underlying the KI‐induced *Nrg1* upregulation and *Gabra2* downregulation, we applied single‐cell regulatory network inference and clustering (SCENIC). This method combines co‐expression analysis with motif enrichment to infer the set of genes directly controlled by transcription factors (TFs), known as a regulon [38]. We found that the regulon activity of TFs predicted to regulate *Gabra2* transcription, such as *Ahr* and *Rarb*, was downregulated in GABAergic neurons in NCOR1^KI/+^ vs. WT mice (Figure S2A). Conversely, the regulon activity of TFs predicted to regulate *Nrg1* transcription, such as *Rfx2*, *Six3*, *Pparg*, and *Taf1*, was upregulated in GABAergic neurons in NCOR1^KI/+^ vs. WT mice (Figure S2A). Consistent with the heatmap, the t‐Distributed Stochastic Neighbor Embedding (t‐SNE) plot of regulon activity revealed that the above differences in regulon activity were more pronounced in GABAergic neurons than in glutamatergic neurons, as WT and NCOR1^KI/+^ GABAergic neurons were more distinctly separated. In contrast, WT and KI glutamatergic neurons showed greater overlap (Figure S2B). The feature plots of binary regulons and their target gene sets activities showed that the regulon activities of *Ahr* and *Rarb* in GABA neurons were reduced in the NCOR1^KI/+^ group compared to WT (Figure S2C), and correlation analysis further showed a robust positive correlation between their regulon activities and the expression level of their predicted target gene *Gabra2* (Figure S2D). Conversely, *Pparg*, *Taf1*, *Rfx2*, and *Six3* displayed increased regulon activities in the NCOR1^KI/+^ group compared to WT within GABA neurons (Figure S2E), which showed a robust positive correlation with the expression level of their predicted target gene *Nrg1* (Figure S2F). Notably, PPARγ (*Pparg*) is a well‐known nuclear receptor whose activity is directly regulated by NCOR1 [39, 40]. Thus, we hypothesize that the loss‐of‐function of *Ncor1* could enhance the activity of *Pparg*, *Taf1*, *Rfx2*, or *Six3*, leading to upregulation of *Nrg1* transcription in hypothalamic GABA neurons. The Nrg1‐ICD then downregulates *Gabra2* expression, potentially through repressing *Ahr* or *Rarb* activity, which elevates the firing activity of GABA neurons and impairs hippocampal synaptic plasticity through the hypothalamic‐hippocampal GABAergic neural projection [15] (Figure S2G).

### 2.5 Enhancing the GABA‐A Receptor Signaling Alleviates Cognitive Dysfunctions in NCOR1^KI/+^ Mice

Disrupted GABA signaling is frequently observed in ASD and other developmental brain disorders [15, 41, 42, 43]. Since our bioinformatics analysis and cell culture studies suggest that upregulated NRG signaling and the subsequent downregulation of GABA‐A receptor signaling might underlie the cognitive impairment in NCOR1^KI/+^ mice, we sought to determine whether the cognitive dysfunction can be mitigated with GABA‐A receptor agonists. SL651498 (SL) is a selective positive modulator of GABA‐A receptors [44, 45]. Allopregnanolone (Allo), approved by the FDA to treat postpartum depression, is a 3α,5α‐progesterone metabolite and a potent allosteric modulator of the GABA‐A receptor [46, 47]. Neither of them has been tested for the therapy of NCOR haploinsufficiency‐related ASD. NCOR1^KI/+^ mice were intraperitoneally (*i.p*.) injected with either SL (2 mg/kg) or Allo (5 mg/kg) 1–2 h before the behavior tests in both training and testing sessions. Compared to the vehicle, both SL and Allo administration effectively ameliorated cognitive deficits in NCOR1^KI/+^ mice in the NOR test (Figure 5A,B), the OLR test (Figure 5C,D), and the 3CH‐SM test (Figure 5E,F), without changing the baseline social interaction behaviors (Figure 5G,H) or baseline explorative behaviors (Figure 5B,D,F). These data demonstrate an indispensable role of GABA signaling in regulating cognition in NCOR1^KI/+^ mice. Importantly, these findings suggest that NCOR‐related cognitive dysfunctions in ASD can be ameliorated by FDA‐approved medicines targeting GABA‐A receptors.

**FIGURE 5:**
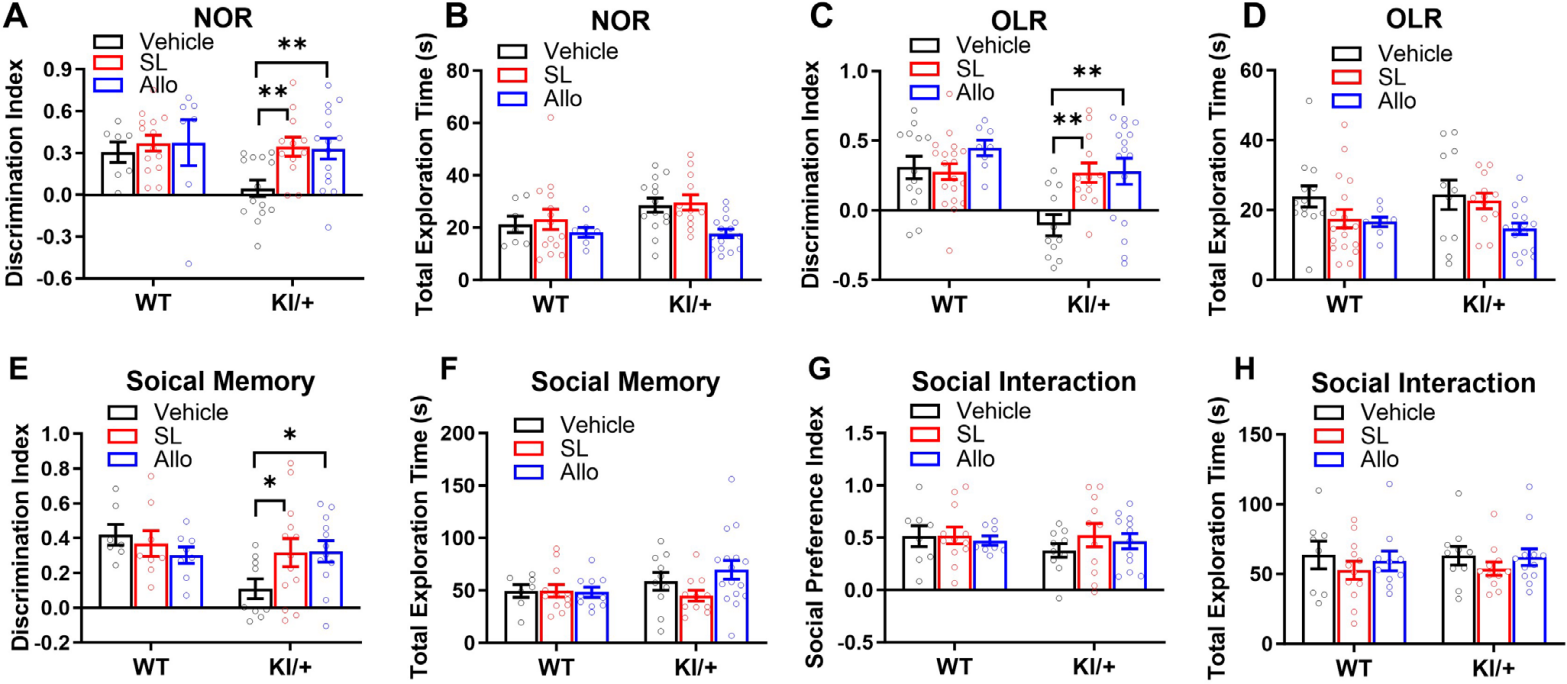
Enhancing the GABA‐A receptor signaling alleviates cognitive dysfunctions in NCOR1^KI/+^ mice. GABA_A_ receptor agonist SL651498 (SL, 2 mg/kg) or positive allosteric modulator allopregnanolone (Allo, 5 mg/kg) or vehicle was administered intraperitoneally (i.p.) to 3‐month‐old male mice daily, beginning 2 h prior to the behavior training and testing. (A, B) Discrimination index and total exploration time in the NOR test. (C, D) Discrimination index and total exploration time in the OLR test. (E, F) Discrimination index and total exploration time in 3CH‐SM. (G, H) Social preference index and total exploration time in 3CH‐SI. The sample size is indicated by the individual data points (n = 7–15 mice per group). Two‐way ANOVA was used, followed by Tukey's post hoc test. Each data point is one mouse. Data are mean ± SEM. ^*^ *p* < 0.05, and ^**^ *p* < 0.01.

### 2.6 Pharmacologic Targeting of NRG1 Signaling Improves Cognition of NCOR1^KI/+^ Mice

To test whether inhibition of NRG1 backward signaling can rescue impaired cognitive function, we used spironolactone, an FDA‐approved medication that blocks NRG1‐ErbB signaling [37]. The drug was given via intraperitoneal injection at a dosage of 50 mg/kg daily, started at least 3 days prior to the first training day and 2 h before the first training session, and continued until the day of the test session. Spironolactone rescued recognition and spatial memory in NCOR1^KI/+^ mice in the NOR test (Figure 6A,B), OLR test (Figure 6C,D), MWM test (Figure 6E–G), and Y‐Maze test (Figure 6H,I). Spironolactone also improved social memory in the 3CH‐SM test (Figure 6J,K) without altering the baseline social interaction in the 3CH‐SI test (Figure 6L,M). Spironolactone did not alter the total exploration time (Figure 6B,D,K,M) or travel distance (Figure 6G,I) in WT or NCOR1^KI/+^ mice in these tests. Spironolactone also did not alter performance in emotion‐related behaviors in the EPM test (Figure 6N,O) and OFA test (Figure 6P,Q). Interestingly, spironolactone did not alter stereotypic behaviors in the marble burying test (Figure 6R), whereas allopregnanolone partially improved them (Figure 6S), suggesting that NRG‐independent mechanisms underlie stereotypic behaviors. RT‐qPCR showed that spironolactone upregulated *Gabra2* expression in the hypothalamus of the NCOR1^KI/+^ mice (Figure 6T). Western blot analysis showed higher levels of NRG1 in the KI/+ mouse brain than in WT (Figure S2H,I), although the magnitude of the effect was likely underestimated due to the presence of non‐GABAergic neurons and non‐neuronal cells in these bulk brain lysates. The apparent molecular weight varies depending on the specific NRG1 protein isoform and its glycosylation status. Importantly, spironolactone disrupted the upregulation of NRG1 levels in KI/+ vs. WT mice. In summary, these results suggest that the NRG1‐GABRA2 signaling pathway regulates cognitive function and identify a potential therapeutic strategy through drug repurposing to improve cognitive function in NCOR loss‐of‐function.

**FIGURE 6:**
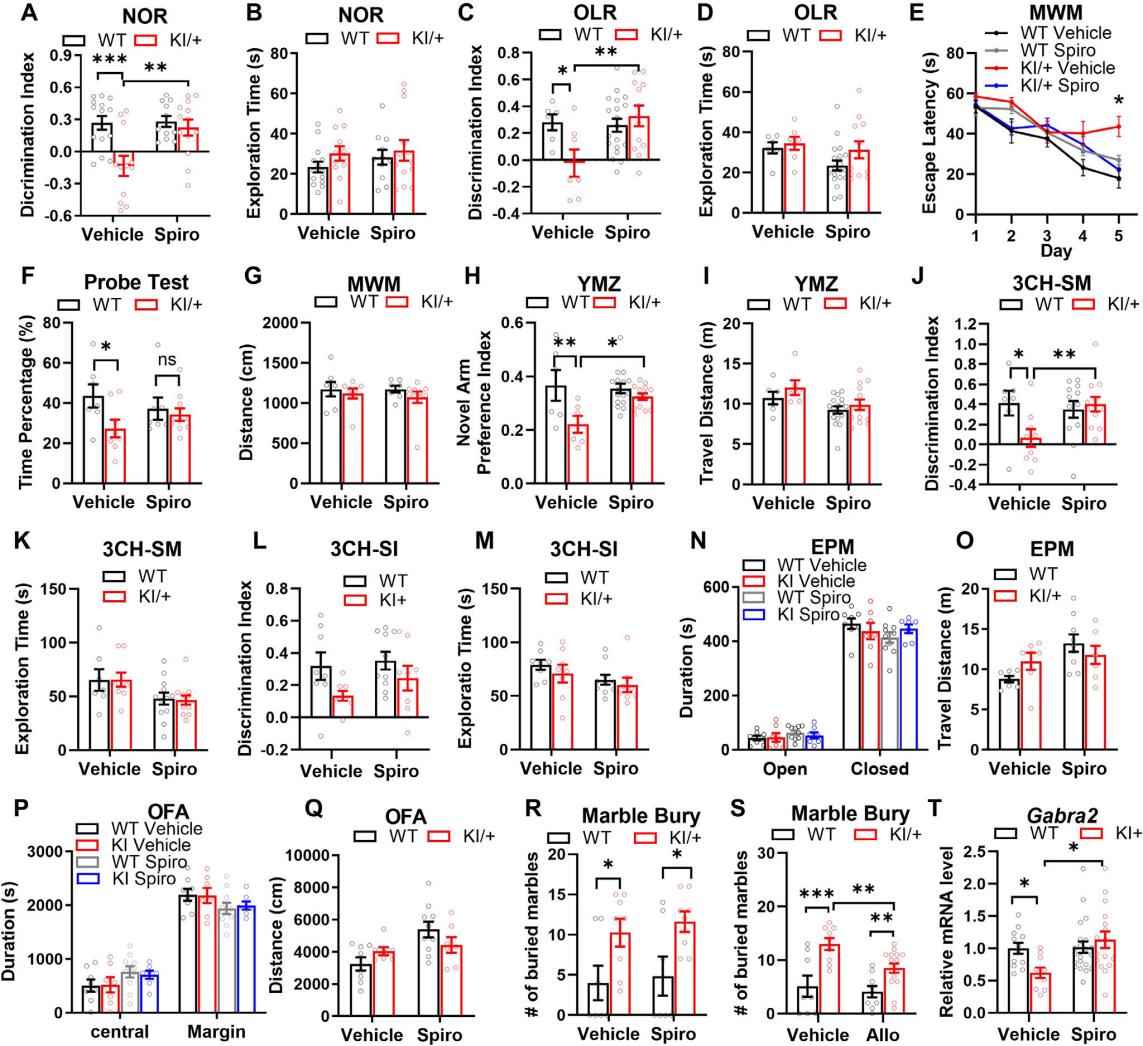
Pharmacologic targeting of NRG1 signaling improves cognition of NCOR1^KI/+^ mice. NRG1‐ErbB antagonist spironolactone (Spiro, 50 mg/kg) or vehicle was administered systemically (i.p.) to 6‐month‐old female NCOR1KI/+ or WT mice daily for 3 days prior to the training day and 2 h before the behavioral training and testing sessions. (A, B) Discrimination index and total exploration time in the NOR test. (C, D) Discrimination index and total exploration time in the OLR test. (E) Escape latency of the Morris water maze (MWM) test during the 5‐day training. ^*^ *p* < 0.05 between KI/vehicle and the other groups by two‐way repeated ANOVA and Tukey's post hoc test. (F) Time percentage spent in the target quadrant zone during the probe test. (G) Total distance traveled in the MWM test. (H, I) Novel arm preference and total travel distance in the Y‐maze test. Data were analyzed by a two‐tailed unpaired t‐test. (J, K) Discrimination index and total exploration time in the 3CH‐SM test. (L, M) Social preference index and total exploration time in the 3CH‐SI test. Data were analyzed by a two‐tailed unpaired t‐test. (N) The ratio of time in open arms to time in closed arms during the EPM test. (O) Trace of exploration activity in the EPM test. (P) Time spent in the central area during the OFA test. (Q) Total travel distance during the OFA test. (R, S) Number of buried marbles during the test period. (T) RT‐qPCR analysis of the effects of Spiro on *Gabra2* expression in the hypothalamus from WT and NCOR1^KI/+^ (KI/+) mice. The sample size is indicated by individual data points. Each data point is one mouse (n = 7–19 per group). Data are mean ± SEM. Two‐way ANOVA, or two‐way repeated ANOVA followed by Tukey's post hoc test, was used unless otherwise indicated. ^*^ *p* < 0.05, ^**^ *p* < 0.01, and ^***^ *p* < 0.001.

### 2.7 NRG1 Signaling Inhibitor Rescues Cognitive Dysfunction in a Patient Carrying an *MECP2* Variant

Spironolactone is widely used to treat fluid build‐up due to heart failure, liver scarring, or kidney disease. We investigated two pediatric patients: a 7‐year‐old girl carrying a *de novo* heterozygous *MECP2* mutation c.397C>T (p.Arg133Cys) who displayed cognitive dysfunction, developmental language disorder, and involuntary hand movements. As a control, we also recruited an age‐matched girl carrying a heterozygous calcium voltage‐gated channel subunit alpha1 A (*CACNA1A*) mutation c.5338C>T (p.R1780X) who displayed delayed intellectual development and language disorder without stereotypic behaviors. Two participants received weight‐adjusted oral spironolactone (2 mg/kg/day, max 50 mg/day) for 3 months. As assessed by the scoring system of the Autism Diagnostic Observation Schedule, second Edition (ADOS2), the *MECP2* patient showed improvement in both social interaction (ADOS2_I: 20%) and restricted/repetitive behavior (ADOS2_II: 25%) after spironolactone treatment (Table 1). The Childhood Autism Rating Scale (CARS) score decreased by 16.7% in the *MECP2* patient (from 36 to 30) compared to only 3.2% in the control (31 to 30). On the Autism Behavior Checklist (ABC), the *MECP2* patient demonstrated improvements in motor skills (40.9%) and self‐care (45.8%), resulting in a 20.8% decrease in total ABC score. The control patient showed much less improvement in all these severity scores.

**TABLE 1:** .

Measures	Scores	MECP2 patient			Control patient		
		Pre‐treatment	Post‐treatment	Δ%	Pre‐treatment	Post‐treatment	Δ%
ADOS	ADOS2_I	5	4	20	2	2	0
	ADOS2_II	12	9	25	7	6	14.29
CARS		36	30	16.67	31	30	3.23
Autism Behavior Checklist	motor skills	22	13	40.91	15	13	13.33
	self‐care	24	13	45.83	14	13	7.14
	total scores	101	80	20.79	92	80	13.04
School‐Age Children's Behavioral Rating Scale	Initiation	15	22	46.67	16	19	18.75
	working memory	20	27	35	20	24	20
	Inhibition	20	29	45	20	21	5
	Organization	12	14	16.67	13	11	−15.38
Vinerland‐3	gross motor	9	11	22.22	11	11	0
	fine motor	14	15	7.14	10	10	0
	adaptive behavior composite total	39	46	17.95	67	69	2.99
	social interpersonal domain	36	52	44.44	72	74	2.78
	adaptive behavior standard score	103	123	19.42	199	210	5.53
Communicative Development Inventory	verb acquisition	5	15	200	193	193	0
	total vocabulary score	21	30	42.86	795	795	0
	phrases understood	25	27	8	27	27	0
	words understood	261	365	39.84	748	763	2.01
	words produced	60	75	25	714	735	2.94
Conners Parent Questionnaire	conduct problems	1.08	0.83	23.15	1.08	1.42	−34.48
	learning problems	2.5	1.5	40	1.75	1.50	14.29
	somatic complaints	1.4	0.4	71.43	1	1	0

ADOS2, Autism Diagnostic Observation Schedule, second Edition. CARS, Childhood Autism Rating Scale.

Δ% = |(post‐treatment‐pre‐treatment)/pre‐treatment| x 100 x D, where D = +1 if the change indicates functional improvement, and D = –1 if the change suggests functional worsening or exacerbation.

In executive function assessed by the School‐Age Children's Behavioral Rating Scale of Executive Function, the *MECP2* patient exhibited improvements across multiple domains: Initiation increased by 46.7% (vs. 18.8% in control), Working Memory by 35.0% (vs. 20.0%), Inhibition by 45.0% (vs. 5.0%), and Organization by 16.7% (vs. −15.4% deterioration in control) (Table 1). These changes represented greater net improvements in the *MECP2* patient across all executive function domains after spironolactone treatment compared to the control.

In motor function domains measured by the Vineland‐3, the *MECP2* patient showed greater gains in both gross motor (22.2% improvement vs. 0% in the control) and fine motor skills (7.1% improvement vs. 0% in the control) after spironolactone treatment compared to the control patient (Table 1). For social and adaptive functioning, the *MECP2* patient achieved more substantial improvement in the Adaptive Behavior Composite standard score (19.4% increase vs. 5.5% in control), Adaptive Behavior Composite total score (17.9% improvement vs. 3.0% in control), and particularly in the Social Interpersonal Relationships domain (SOC), where the *MECP2* patient showed 44.4% improvement compared to only 2.8% in the control (Table 1). In language development assessed by the Communicative Development Inventory (CDI), the *MECP2* patient exhibited increases in verb acquisition (200% improvement vs. 0% in control), total vocabulary score (42.9% improvement vs. 0% in control), phrases understood (8.0% improvement vs. 0% in control), words understood (39.8% improvement vs. 2.0% in control), and words produced (25.0% improvement vs. 2.9% in control). Regarding behavioral measures on the Conners Parent Questionnaire, the *MECP2* patient showed greater improvement in Conduct Problems (23.1% vs. −34.5% in the control) and Learning Problems (40.0% vs. 14.29% in the control) after spironolactone treatment compared to the control patient (Table 1). Somatic complaints in the *MECP2* patient improved after spironolactone treatment, whereas those in the control did not change. The treatment was well tolerated, with no adverse events reported during the intervention period (Table 2). In summary, this case study provides the proof‐of‐concept evidence that spironolactone may lead to multi‐domain improvements in core autism symptoms, motor abilities, social function, language skills, and behavior in patients carrying NCOR/MECP2‐related mutations.

**TABLE 2:** .

Measures	Scores	MECP2 patient			Control patient		
		Pre‐treatment	Mid‐treatment	Post‐treatment	Pre‐treatment	Mid‐treatment	Post‐treatment
Complete blood count (CBC)	WBC	5.72	7.81	8.02	4.78	5.04	5.08
	RBC	4.7	4.79	5.08	4.22	4.42	4.36
	NEU	2.03	4.1	4.71	2.17	1.99	2.47
	HB	136	140	147	120	118	124
	PLT	244	248	278	255	165	203
Urine	pH	9	5.5	5	5.5	6	5
	protein	+‐	+‐	—	—	—	—
	Glucose	—	—	—	—	—	—
Blood biochemistry	ALT	13	10	10	11	9	11
	AST	29	26	23	27	27	28
	GGT	9	8	9	11	10	10
	Urea	4	6.58	4.1	3.62	4	3.86
	Creatinine	33	31	31	39	35	36
	eGFR	168.5	172.1	172.1	158.5	165.4	178.4
	K	4.49	4.21	4.49	4.26	4.18	4.11
	Na	141	143	140	142	138	139
	Ca	2.54	2.56	2.59	2.36	2.38	2.37
Cardiac examination	SBP	87	89	91	90	95	87
	DBP	60	59	62	58	61	59
	ECG	Normal	Normal	Normal	Normal	Normal	Normal

WBC: White blood cell count. RBC: Red blood cell count. NEU: Neutrophils. HB: Hemoglobin. PLT: Platelets. pH: Potential of hydrogen. ALT: Alanine aminotransferase. AST: Aspartate aminotransferase. GGT: Gamma‐glutamyl transferase. eGFR: estimated glomerular filtration rate. K: Potassium. Na: Sodium. Ca: Calcium. SBP: Systolic blood pressure. DBP: Diastolic blood pressure. ECG: Electrocardiogram. Mid‐treatment: 1.5 months after the start of the treatment. Post‐treatment: 3 months after the start of the treatment. +‐: weakly positive. ‐: negative.

## 3 Discussion

Our study and others previously found the association of *NCOR1/2* genetic variants with ASD and intellectual disability [15, 19]. However, it is still unknown whether a heterozygous mutation only in *NCOR1* or *NCOR2* is adequate to induce ASD‐related phenotypes. Here, we established the causal role of a heterozygous *NCOR1* loss‐of‐function mutation in intellectual disability through two new genetically modified mouse models: heterozygous *Ncor1* knockout and c.2182+2T>G mutation KI mice. Both mouse strains exhibited cognitive impairments and stereotypic behaviors similar to those observed in humans. FDA‐approved drugs targeting NRG1 or GABA‐A signaling efficiently rescued cognitive defects in these models. Collectively, this study elucidates NCOR haploinsufficiency‐related cognitive dysfunctions and establishes a new NCOR^KI/+^ mouse model as a useful tool to investigate the molecular mechanisms and innervation strategies underlying *NCOR* mutation‐related ASD.

The snRNA‐seq demonstrates that GABA‐A receptor subunits are downregulated in GABAergic neurons, in line with our previous finding about the critical role of GABA‐A receptors in maintaining the excitatory and inhibitory (E/I) ratio of LH^GABA^ neurons and cognitive functions [15]. The E/I imbalance is a recurrent topic in ASD etiology [48, 49]. Genetic ablation of ASD‐associated genes Sh3rf3, Cntnap2, and Tsc2 impairs excitatory synaptic transmission and increases the excitability of inhibitory neurons, leading to disruption of cortical E/I balance and autistic‐like behaviors in mice [50, 51, 52]. Increased E/I balance is also detected by magnetic resonance spectroscopy in ASD patients, implying that the E/I imbalance should be brain‐region‐, disease‐subtype‐, or stage‐specific [53]. Additionally, copy number variation of GABA receptor genes was associated with autism [41, 54, 55], and downregulation of GABA receptor subunits has been observed in the postmortem brain tissue of autistic individuals [56, 57]. GABA‐A receptors are decreased in the dorsal hippocampus of idiopathic (BTBR strain) and genetic (Fmr1 and Shank3 mutants) mouse models of ASD in adulthood [58]. Clonazepam, an FDA‐approved GABAA receptor‐positive allosteric modulator, improves sociability in *Scn2a‐*deficient ASD‐like mice and normalizes E/I ratio in human brain organoids carrying an autism‐associated *SCN2A* nonsense mutation [59]. GABA agonists were also studied in the context of ASD. For example, GABA‐B agonists alone or in combination with GABA‐A agonists showed beneficial effects in prenatal valproic acid (VPA) animal models of ASD [60, 61]. However, clinical studies of arbaclofen, a GABA‐B receptor agonist, have yielded largely negative or inconclusive results in ASD patients [62, 63], highlighting the substantial biological heterogeneity within ASD and suggesting that therapeutic responses may depend on the underlying etiology. The present study is the first study to test GABA‐A receptor agonists in an NCOR‐related ASD model. Our rescue experiments showed that enhancing GABA signaling via GABA‐A receptor agonists in adult NCOR1^KI/+^ mice efficiently ameliorated cognitive dysfunction and stereotypic behaviors. These results suggest that cognitive deficits and repetitive behaviors in *NCOR1* mutant mice are unlikely due to irreversible developmental anomalies.

Our study suggests a role of NRG in ASD etiology. NRG signaling is involved in many neurodevelopmental disorders and plays roles in glial cell development, axon myelination, neurite growth, synapse formation, and synaptic plasticity [22]. NRG1 has multiple splicing sites, generating various isoforms with distinct amino‐terminal regions. These isoforms differ in their levels and expression patterns across multiple tissues, including the brain. NRG1 signaling is thought to have neurotrophic and neuroprotective functions. Intranasal replenishment of recombinant NRG1 protein effectively reduced neuron death and ameliorated motor function deficits in mice after traumatic brain injury [34]. In a mouse epilepsy model, stereotaxic injection of NRG1 protein into the right lateral ventricle delayed the onset and decreased the incidence of seizures by increasing the intrinsic excitability of fast‐spiking parvalbumin‐positive interneurons [64]. However, postmortem brain samples from schizophrenia patients also showed increased *NRG1* mRNA levels [65, 66, 67]. Overexpression of *Nrg1* in GABAergic interneurons in mice resulted in cortical disinhibition and schizophrenia‐like behaviors [68]. Thus, either NRG1 deficit or overactivation can lead to pathogenetic changes in the neuropsychiatric system. The hypothalamic GABAergic neurons of NCOR1^KI/+^ mice showed increased *Nrg1* expression, and pharmacological inhibition of NRG1‐ErbB interaction rescued cognitive behaviors. Therefore, NCOR1 contributes to the delicate regulation of NRG1 signaling. Since inhibiting NRG1 signaling ameliorates cognitive deficits but not stereotypic behaviors, our results suggest that NCOR‐related cognitive dysfunction and stereotypic behaviors may not share the same etiology.

Our findings suggest that increased NRG1 expression regulates *Gabra2* expression through the noncanonical backward pathway, although the exact mechanism by which NRG1‐ICD regulates gene expression remains elusive. NRG1 consists of an N‐terminal region, an EGF‐like domain, a transmembrane domain, and an intracellular domain [32]. The bioactive extracellular domain of NRG1 binds to ErbBs, initiating canonical or noncanonical forward signaling. In addition to forward signaling, NRG1 can also influence transcription through backward signaling, in which the NRG1 protein is cleaved, and the ICD fragment enters the nucleus to regulate gene expression [35]. Interaction between NRG1 and ErbB receptors can target NRG1‐ICD to the nucleus in neurons, thereby regulating gene expression [35]. Overexpression of ICD but not the EGF‐containing domain (ECD) decreases neural stem cell proliferation and increases neuronal and oligodendroglial differentiation [69]. NRG1‐ICD forms a complex with a zinc‐finger transcription factor Eos to induce endogenous PSD‐95 expression in vivo, providing a molecular basis for activity‐dependent synaptic plasticity [70]. The missense mutation (V321L) in the NRG1 transmembrane domain that disrupts nuclear back signaling with minimal effects on forward signaling is associated with schizophrenia [71]. The V321L mutation, which impairs NRG1‐ICD nuclear translocation, alters gene expression in the mouse hippocampal dentate gyrus. V321L mice show reduced stem cell proliferation, altered cell cycle dynamics, defects in fate specification, and abnormal dendritic dysmorphogenesis [72]. Spironolactone may disrupt the NRG1 backward signaling, thereby partially rescuing *Gabra* expression. Both spironolactone and allopregnanolone are FDA‐approved drugs. Spironolactone is a diuretic medication used to treat fluid build‐up due to heart failure, liver scarring, kidney disease, high blood pressure, or low blood potassium that does not improve with supplementation. It is also an antiandrogen and can be used to treat early puberty in boys, acne, and excessive hair growth in women. Allopregnanolone is used to treat postpartum depression. Our findings suggest that spironolactone and allopregnanolone or related derivatives could be repurposed to alleviate the intellectual disability of ASD patients carrying NCOR‐related mutations.

There are several limitations in the present study. (1) Although we found NRG1‐ICD inhibited the expression of *Gabra2*, other mechanisms, including forward ErbB downstream signaling, may also participate in the regulation process. (2) Spironolactone has many roles, including as a mineralocorticoid receptor (MR) antagonist and an androgen receptor (AR) antagonist. We cannot completely rule out a contribution from other signaling pathways to the behavioral rescue effects of spironolactone. (3) The clinical study only includes a single patient without blinding or a placebo control, so the possibility of developmental progression or observer bias may be present. (4) The patient carries a single MECP2 mutant, but not NCOR1. Although NCOR forms a complex with MECP2 to regulate gene expression in neurons, we did not test the direct effects of the MECP2 mutation on the NRG1/GABRA2 axis in the patient. Therefore, the current evidence provides clinical proof‐of‐concept rather than therapeutic validation in NCOR‐related intellectual disability.

## 4 Method

### 4.1 Animals

NCOR1^+/−^ mice were generated by crossing NCOR1^loxP/+^ mice (PHENOMIN, Institut Clinique de la Souris (ICS), CNRS, INSERM, University of Strasbourg, France) with β‐actin‐*Cre* transgenic mice (stock # 019099, The Jackson Laboratory). All mice were from a C57BL/6 genetic background. The sex and age of the mice for each experiment were indicated in the figure legends. No drastic sex differences were observed in the phenotypes of NCOR1^+/−^ and NCOR1^KI/+^ mice, although the phenotypic effects of NCOR1 disruption appeared to be more robust in male mice or older female mice compared to young female mice. However, cohort‐to‐cohort variability may also contribute to these differences. WT littermates were used as control mice. All tests were repeated at least two times. Four mice were housed per cage in a 12 h light/12 h dark (7 am to 7 pm) facility with free access to water and food. Animals were grouped by sex, age, and genotype. All animal experiments were conducted in accordance with the institutional and national guidelines for the care and use of laboratory animals and were approved by the Institutional Animal Care and Use Committee (IACUC) of Baylor College of Medicine under protocol (AN‐6583). All procedures were performed in accordance with applicable animal welfare regulations and guidelines (Animal Welfare Assurance Number: D16‐00475).

The NCOR1 (c.2182+2T>G) knock‐in (NCOR1^KI/+^) mice were generated by Shanghai Model Organisms Center, Inc. (SMOC, Shanghai, China). Briefly, the targeting vector was constructed by the infusion method which contains a 3.0 kb 5’ homologous arm followed by the intron 18 of *Ncor1* containing the mutation c.2182+2T>G, PGK‐Neo‐polyA cassette, 2.7 kb 3’ homologous arm, and MC1‐TK‐polyA gene as a negative selection marker, a FRT‐pGK‐Neo‐FRT selection cassette. JM8A3 embryonic stem (ES) cells were transfected with the linearized targeting vector and selected with 250 µg/mL G418. The clones were screened by PCR and Sanger DNA sequencing for correct targeting into the *Ncor1* genomic locus. Two correctly targeted ES clones were injected into C57BL/6J blastocysts to generate chimeric mice, which were further crossed with Flp mice to excise the pGK‐Neo selection cassette, and the progeny were screened by PCR and Sanger DNA sequencing. Heterozygous knock‐in mice lacking pGK‐Neo were bred with C57BL/6J mice to generate NCOR1^KI/+^ mice. Tail tissue from NCOR1^KI/+^ was incubated in 80% Proteinase‐K for 6 h before the purification of genomic DNA and PCR validation with specific primers (forward 5′‐GGAAACCCCGTGAGGAAC‐3′, reverse 5′‐ATACAAAGAGGCATACAAA GATA‐3′). The genotype of NCOR1^KI/+^ mice was determined by sequencing the PCR product of 725 bp with the forward primer.

### 4.2 Behavioral Tests

Behavior tests were performed between 12 and 6 pm in a dim light environment (300 lumens) unless stated otherwise. Mice were given a 2‐week interval to recover from the previous behavioral test before running the next behavioral test. Mice were not subjected to more than four behavioral tests. For the Novel Object Recognition (NOR) test, a three‐day protocol was followed [15]. Briefly, the objects used were built from Lego and were provided by the Neurobehavioral Core at Baylor College of Medicine. A small arena (22 cm × 44 cm) was used to facilitate object exploration and reduce the time needed to habituate mice to the area and environment. A white screen surrounded the arena on three sides to limit spatial information and prevent spatial biases. During the training session, two identical objects were placed on the right and left sides of the arena. Mice were placed in the center of the arena and allowed to explore freely for 5 min each day for two days. After each training session, mice were returned to their home cages. 24 h after the second training session, one of the objects was replaced with a novel object of a different color and shape. The mice were then allowed to freely explore the entire arena for 5 min. Animal behavior during the training and test session was tracked by a top camera and analyzed by ANY‐maze software (Stoelting). The discrimination index was calculated as (exploration time on the novel object‐exploration time on the familiar object) / (exploration time on the novel object + exploration time on the familiar object). Sniffing, touching (>1 s), and staring at the objects were judged as exploration behaviors.

For the Object Location recognition (OLR) test, a two‐day protocol was followed with minor modifications [73]. Briefly, the objects used were built from Lego and were provided by the Neurobehavioral Core at Baylor College of Medicine. On the first day, mice were first exposed to an empty small arena (44 cm × 44 cm) surrounded by a white screen on three sides for 10 min. Following this, two identical objects were placed into the same arena, and the mice were trained to explore it for another 10 min. After the training session, the mice were returned to their home cages. 24 h later, one of the objects was moved to a new location in the same arena. The mice were then allowed to freely explore the entire arena for 10 min. Animal behavior during the training and test session was tracked by a top camera and analyzed by ANY‐maze software (Stoelting). The discrimination index was calculated as: (exploration time on the object at the new location‐exploration time on the object at the familiar location) / Total exploration time on the objects at both the new location and original location. Sniffing, touching (>1 s), and staring at the objects were judged as exploration behaviors.

The Y‐Maze short‐term spatial memory was measured according to the established procedure with minor modifications [74]. The Y‐maze, made of white opaque acrylic, had three arms (height: 20 cm; length: 30 cm; width: 8 cm) positioned 120 degrees apart. Spatial cues were placed on the walls aligned with the axes of the familiar and novel arms. The paradigm consisted of a 5‐min encoding trial during which one arm was blocked off, followed by a 1‐h intertrial interval, and then a 5‐min retrieval session. The start arm remained the same in both the encoding and retrieval trials, while the exposed arm during the encoding trial was considered the familiar arm, and the blocked arm was considered the novel arm. The apparatus was wiped with 30% ethanol and dried between each mouse to remove odor cues. The discrimination index was calculated as the comparison between the times spent in the novel and familiar arms during the retrieval paradigm.

The three‐chamber social memory (3CH‐SM) and social interaction (3CH‐SI) tests were performed in a three‐chamber (Crawley) apparatus (60 × 40 × 25 cm) that consisted of three chambers (left, center, and right) of equal size with 10 × 5 cm openings between the chambers, slightly bigger than those in the previous report. Mice were given two consecutive 10‐min tests: the first measured baseline activity in the apparatus, and the second test measured the sociability of the test mouse given a choice of exploring a chamber containing another mouse under a mesh pencil cup or a chamber containing a novel object under a mesh pencil cup. A camera and the ANY‐maze software program were used to track the position of the mouse in the three‐chambered box, while the experimenter scored the approaches to the object or partner mouse using a wireless keyboard. Partner mice (sex‐, age‐, and weight‐matched) were purchased from the Center for Comparative Medicine (CCM) at Baylor College of Medicine and habituated to the mesh pencil cups in the apparatus for 1 h per day for 2 days before the day of testing. Partner mice were used up to 3 times per day, with 1 test per day. To test social memory, a third test was conducted for 10 min in the same chamber, with one chamber containing the familiar mouse under a mesh pencil cup and the other containing another novel mouse. The memory is quantified using the discrimination index, as discussed in the NOR test.

The Morris water maze (MWM) test was performed as described previously, with modifications [15]. During the training session, a transparent rescue platform was submerged in the painted water (0.5–1 cm) and placed in a fixed position in the pool. On the first day of training, mice were first allowed to stand on the platform for 10 s. After that, mice were gently placed into the water maze, facing the pool wall, and allowed to freely explore the entire maze for 1 min. Mice were then guided to the rescue platform if they did not find it. Mice were allowed to rest on the platform for 10 s, then retrained from a different start position using the same procedure. After four training trials, they were dried using a paper towel and returned to their home cages. 24 h later, the mice were trained again using the same procedure, without the initial habituation session. Mice were trained for 5 consecutive days. At the end of the fourth trial on day 5, mice were returned to home cages for recovery. 1 h later, mice were put back into the water maze for 1 min, where the platform had been removed. Mouse behaviors were videotaped and analyzed by the Noldus EthoVision XT. The MWM was virtually divided into four quadrants. The rescue platform was located in the target quadrant. Escape latency was defined as the time spent before finding and climbing onto the platform. Escape latency during the 5‐day training sessions served as an independent measurement of spatial learning and memory.

For the Elevated Plus Maze (EPM) test, we used a plus‐shaped platform elevated 40 cm above the floor. Two opposite arms of the maze were walled (15 cm high), whereas the other two arms were open, with a 5 mm‐high ridge to prevent falling. Each arm was 8 cm wide and 25 cm long. The test lasted 10 min and began by placing a mouse in the center of the maze, facing one of the two open arms in a bright environment (900 lumens). An overhead camera and the ANY‐maze software were used to track the mouse's position in the elevated plus maze. The number of visits to the open arms and the time spent on them were used as measures of anxiety. The open‐field arena (OFA) test was performed using the Versamax animal activity monitoring system, which is equipped with infrared photo beams to detect activity in the horizontal and vertical directions. Mice were placed in the center of the open‐field arena (40 cm × 40 cm × 30 cm) and allowed to explore for 45 min. The locomotor activity and location of the mice were scored automatically by VersaMax software. The percentage of time spent and/or distance traveled in the center area measures anxiety levels. The light–dark test was performed in a box (42 cm × 20 cm × 25 cm) that contained a dark area (one‐third of the box) and a light area (two‐thirds of the box) connected by a small opening to allow mice to move from one area to the other. The test lasted for 10 min and started by placing a mouse in the bright area. The activity and location of the mouse were scored automatically by VersaMax software. The number of transitions between dark and light zones and the time spent in the light and dark areas served as the index of anxiety^53^. The Rotarod test was performed using an accelerating rotarod (UGO Basile)^55^, with minor modifications. Mice were placed on a rotating drum that accelerated from 4 to 40 rpm over 5 min, without accommodation training. We recorded the time spent walking on top of the rod before falling off, or hanging on and riding completely around it. The rod was only 20 cm above the platform, so mice were not injured when falling off the rod. Mice were given two trials per day for four consecutive days, with a maximum time of 5 min per trial and a 30–60 min inter‐trial rest interval.

The Marble bury test was conducted following the previous study [75] to assess the stereotypic behaviors. This test is a sensitive measure of stereotypic behaviors in mice and a rapid way to assess repetitive behavior in large groups. The mice were placed in clean Allentown mouse cages (27 × 16.5 × 12.5) filled with corncob bedding, with 20 black glass marbles (15 mm diameter) placed in an equidistant 4 × 5 pattern on the surface [76]. The mice were tested for 30 min in a room with a background white noise generator (55 dB). We recorded the number of marbles that were buried, defined as those with their entire surface covered by bedding material. For the grip strength test, forelimb grip strength was measured using a Grip Strength Meter (Columbus Instruments). Each mouse was held by the tail and allowed to grasp a trapeze bar with its forepaws. Once the mouse grasped the bar with both paws, it was gently pulled away from the bar until it released its grip. The digital meter displayed the level of tension exerted on the bar in gram‐force (gf).

### 4.3 Plasmids, Cell Culture, Western Blot, and Immunofluorescence

Neuro‐2a cells were kindly provided by the Stem Cell Bank, Chinese Academy of Sciences, and cultured in Eagle's Minimum Essential Medium (Gibco) supplemented with 10% fetal bovine serum FBS (Gibco) in the incubator at 37°C and 5% CO_2_. The cDNA sequences of mouse *Nrg1* (NM_001364421) encoding pro‐neuregulin‐1, membrane‐bound isoform 2, and NRG1‐ICD (NM_001364421(279‐645aa) were cloned into the GV657 vector with CMV enhancer‐MCS‐3flag‐polyA‐EF1A‐zsGreen‐sv40‐puromycin cassette by restriction enzyme BamHI and KpnI. Recombinant NRG1 and NRG1‐ICD plasmids were validated both by PCR and Sanger DNA sequencing. For Western blot, total cell lysates or brain tissue lysates were resolved by Tris‐glycine SDS‐PAGE, transferred to polyvinylidene difluoride (PVDF) membranes, and blotted with antibodies against Flag (F1804, MBL), β‐actin (81115‐1‐RR, Proteintech), Nrg1 (A0687, Abclonal), NCOR1 (A301‐146A, Bethyl), GAPDH (#2118L, CST), and Hsp90(4874S, CST). After incubation with the appropriate secondary antibodies, the membranes are exposed using the Dual‐ECL Kit (FD8020, FuDe) and a Tanon 3500 Gel Imaging System.

Mice were anesthetized for transcardiac perfusion with cold PBS and 4% paraformaldehyde. Overnight‐fixed brains were immersed in 30% sucrose, and embedded in the O.C.T. compound and frozen in isopentane. Coronal brain sections (30 µm) were prepared on the Leica CM1850 cryostat slicer. The coronal sections corresponding to Bregma: −1.6 mm to −0.5 mm were collected. Anti‐GAD67 antibodies (ab75712, Abcam, 1:500), anti‐cFos (#226308, SYSY, 1:1000), Goat anti‐Guinea Pig IgG (H+L) (A‐21450, Invitrogen. 1:1000), and Alexa Fluor 568 AffiniPure Donkey Anti‐Chicken IgY (IgG) (H+) (#703‐575‐155, Jackson ImmunoResearch Lab, 1:1000) were diluted in PBS blocking buffer separately before use. Floated brain sections were incubated with the first antibodies at 4°C overnight. After that, sections were washed for 3 times in PBS at room temperature and further incubated with second antibodies tagged with fluorescence group for 1 h at room temperature. These sections were then washed 3 times in PBS at room temperature, counterstained with Hoechst, and sealed with the coverslip.

#### 4.3.1 snRNA‐seq and RT‐qPCR

Single‐nuclei RNA‐seq was performed using the nuclei extracted from the hypothalamus in NCOR1^KI/+^ mice and their littermate wild‐type control mice (n  =  2 in each group). In brief, the mouse hypothalamus was homogenized in ice‐cold Nuclei EZ Lysis buffer (NUC‐101; Sigma–Aldrich) supplemented with protease inhibitor (5892791001; Roche) and RNase inhibitor (N2615; Promega and AM2696; Life Technologies). De‐fragment was performed with Miltenyi (Debris Removal Solution), after which the pellet was resuspended in Nuclei Suspension Buffer (1x PBS,0.07% BSA, and 0.1% RNase inhibitor) and filtered through a 20‐µm cell strainer (43‐50020‐50; pluriSelect). The nuclei were counted and adjusted to 700–1200 cells/µl. Single‐nuclei suspensions were loaded to 10x Chromium to capture 8000 single cells according to the manufacturer's instructions of the 10X Genomics Chromium Single‐Cell 3’ kit (V3). The cDNA amplification and library construction steps were performed according to the standard protocol. Libraries were sequenced on an Illumina NovaSeq 6000 sequencing system (paired‐end multiplexing run,150 bp) by LC‐Bio Technology Ltd. (Hangzhou, China) at a minimum depth of 20,000 reads per cell. Sequencing results were demultiplexed and converted to FASTQ format using Illumina bcl2fastq software (version 5.01).

For RT‐qPCR analysis, primers were as follows: *Gabra2* (forward: GGA CCC AGT CAG GTT GGT G, reverse: TCC TGG TCT AAG CCG ATT ATC AT): *Nrg1* (forward: ATG GAG ATT TAT CCC CCA GAC A, reverse: GTT GAG GCA CCC TCT GAG AC); *Ncor1* (forward: GTT TCT GCC CAA GAG GAT GA, reverse: CTC CAC GCT TTC CCT TTC T). Primers for cellular experiments: *Gabra2* (forward: CTA TGC GGT AGC TGT TGC CA, reverse: CTT CTT GTT CGG TTC TGG CG); *Nrg1* (forward: GAG TCA GCT GCA GGC TCC AAG C, reverse: GTG ATG TTG GCA GAG GCA CTG TC); *Nrg1‐ICD* (forward: ATA GTG GAG GAT GAG GAA TAC G, reverse: CAG AAC TTG GGT TGC TGT CC); *Gapdh* (forward: GGT TGT CTC CTG CGA CTT CA, reverse: TGG TCC AGG GTT TCT TAC TCC).

### 4.4 Bioinformatic Analysis of snRNA‐seq Data

We mapped raw reads to the reference genome using STAR (Spliced Transcripts Alignment to a Reference) with default settings, obtaining mapped reads with valid barcodes and unique molecular identifiers (UMIs). Quality control was applied by removing low‐quality nuclei (less than 200 genes) (Seurat v5.1.0) [77] and removing genes detected in less than 3 nuclei. After quality control, the output expression matrix was normalized with the “NormalizeData” function and scaled by the “ScaleData” function. The batch effect was corrected with the harmony package (v 1.2.1) using the“RunHarmony” function in the Seurat object.

For dimension reduction and clustering, the top 2000 variable genes were used for downstream principal component analysis (PCA) before dimensionality reduction. The top 10 principal components were then input for Uniform Manifold Approximation and Projection (UMAP) dimensionality reduction. We identified clusters by applying the FindCluster function (resolution = 0.5). Based on the expression of canonical marker genes, unsupervised‐defined cell clusters were classified into different cell types. For differentially expressed genes (DEGs) analysis, DEGs for identifying marker genes of each cell type were performed using Seurat's FindMarkers function (Wilcoxon Rank Sum test, min.pct = 0.25, logfc.threshold = 0.25, adjust p value<0.05). For comparisons between the two groups, the limma R package(v 3.60.4) was used to identify DEGs. For integration with GWAS hits, we used the reference gene set (Autism, EFO0003758) in the GWAS Catalog (https://www.ebi.ac.uk/gwas/efotraits/). Enrichment scores of GWAS hit‐Autism were calculated with the AddModuleScore function of Seurat with default parameters.

SCENIC (v 1.3.1) [38] was applied to predict the potential gene regulatory networks (GRNs) within neurons of WT and Ncor1^nKI+^ groups. Regulons that consist of upstream transcription factor (TF) and their target genes were inferred using GENIE3 (v 1.26.0), a tree‐based machine‐learning algorithm that models non‐linear expression dependencies between TFs and putative downstream genes from single‐cell expression profiles. Briefly, GENIE3 was applied to the filtered, log_2_(x+1)‐transformed expression matrix to generate a ranked list of candidate target genes for each TF, based on their expression covariation across all neurons. These TF–target pairs were then assembled into preliminary co‐expression modules, which served as the input for subsequent motif‐based filtering. To retain only biologically plausible, direct regulatory interactions, the candidate co‐expression modules were filtered using RcisTarget (version 1.24.0), which evaluates the enrichment of TF‐specific DNA‐binding motifs in the regulatory regions of predicted target genes. Genomic regions for TF‐motif enrichment analysis were restricted to a 10 kb window centered on the transcription start site (TSS). The mm10 mouse genome assembly was used, with motif databases obtained from https://resources.aertslab.org/cistarget/. Only target genes exhibiting significant enrichment of the corresponding TF's binding motif were preserved as high‐confidence direct targets. Putative indirect or spurious targets with negligible motif enrichment were discarded to improve the specificity of the resulting regulons. Each validated regulon thus consists of a single upstream TF and its high‐confidence, motif‐supported target genes. The activity of each refined regulon was then quantified at single‐nucleus resolution using AUCell (version 1.26.0). For each cell, AUCell calculates an Area Under the Curve (AUC) score by evaluating the enrichment of regulon target genes within the top‐ranked expressed genes of that cell. This approach provides a continuous, threshold‐independent measure of regulon activity, enabling reliable comparison of regulatory program activation across individual neurons and between experimental groups. These comparisons were visualized with the pheatmap (v 1.0.12) and Rtsne (v 0.17) packages. All codes, parameters, and step‐by‐step annotated analysis protocols for SCENIC analysis are available on its official GitHub repository (https://github.com/aertslab/SCENIC?tab=readme‐ov‐file). No additional code was implemented, and no parameters were modified in our analysis.

For cell‐cell communication analysis, the R package CellChat (v2.1.2) [26] was used to study the interaction networks between different cell types within the hypothalamus snRNA data. Ligand‐receptor interactions were considered valid and were expressed in at least 10 cells. Based on the method named “trimean”, the CellChat algorithm calculates aggregated ligand‐receptor interaction scores and then weighted scores based on the number of each cell type, defined as interaction strength. First, the core analysis object was constructed using the *createCellChat* function, with input consisting of normalized gene expression matrices and pre‐annotated cell‐type metadata to ensure alignment with established cellular identities. The *subsetDB* function was then used to filter the human L‐R interaction database to retain only secreted signaling pathways, focusing downstream analysis on paracrine and autocrine communication. Subsequent preprocessing steps included applying the subsetData function to reduce computational complexity, along with the *identifyOverExpressedGenes* and *identifyOverExpressedInteractions* functions to enrich for cell‐type‐specific L‐R pairs and improve the specificity of network predictions. Next, intercellular communication probabilities were calculated using the *computeCommunProb* function, and low‐confidence interactions were eliminated via the *filterCommunication* function to retain only robust signaling events. Pathway‐level signaling activity was aggregated from individual L‐R pair probabilities using the *computeCommunProbPathway* function, and the global cell‐cell communication network was further consolidated with the *aggregateNet* function by summing interaction counts and signaling strengths across all cell populations. For network visualization, the *netVisual_aggregate* function generated hierarchical plots and circle plots for individual signaling pathways, while the *netVisual_bubble*, *netVisual_chord_cell*, and *netVisual_chord_gene* functions produced bubble plots and chord diagrams to display multi‐pathway or multi‐L‐R pair‐mediated communication. The *netAnalysis_contribution* function quantified the relative contribution of each L‐R pair to its parent signaling pathway, and the *plotGeneExpression* function was used to visualize the expression distribution of key signaling genes across cell types via violin and dot plots.

To characterize the functional roles of distinct cell populations in the communication network, the *netAnalysis_computeCentrality* function was applied to calculate network centrality scores, identifying dominant signal senders, receivers, mediators, and regulators. The *netAnalysis_signalingRole_scatter* and *netAnalysis_signalingRole_heatmap* functions were then used to visualize the outgoing/incoming signaling capacity of each cell group and pinpoint the major pathways driving these signals. Global communication patterns were identified using the *identifyCommunicationPatterns* function, with results illustrated via alluvial river plots (*netAnalysis_river*) and dot plots (*netAnalysis_dot*) to reveal coordinated signaling between cell populations and pathways. Finally, functional and structural similarities among signaling pathways were quantified using the *computeNetSimilarity* function. Manifold learning (*netEmbedding*) and clustering (*netClustering*) were performed on these similarity metrics, and the netVisual_embedding function was used to generate two‐dimensional projections of pathway clusters, enabling interpretation of pathway redundancy and specificity based on their communication profiles. The CellChat algorithm can compare 2 or more snRNA objects using the function “*mergeCellChat*” and generate a comparative score for the given cell types. All codes, parameters, and step‐by‐step annotated analytical procedures for SCENIC analysis are accessible from the official GitHub repository (https://github.com/jinworks/CellChat?tab=readme‐ov‐file). No additional code was implemented, and no parameters were modified.

### 4.5 Clinical Study

This single‐arm, exploratory clinical trial evaluated the efficacy of spironolactone in two children. The two patients completed a 3‐month treatment period. Results were evaluated at baseline, 1.5 months, and 3 months. This study was prospectively registered at ClinicalTrials.gov (NCT06678685) and approved by the Scientific Review Board and the Institutional Ethics Committee of Qilu Hospital (approval number QLCR20230545). Written informed consent was obtained from the participants’ legal guardians before enrollment. The consent form informs participants and their families about the purpose and rationale of the clinical trial, the study procedures and timeline, eligibility criteria, potential benefits and possible outcomes, foreseeable risks and adverse effects, and how adverse events will be monitored and managed. It also explains the cost and compensation arrangements, alternative treatment options, and the voluntary nature of participation, including the participant's right to withdraw from the study at any time without penalty or loss of medical care. In addition, the consent form describes how personal and medical information will be protected to maintain confidentiality and privacy, how data may be used in future research if applicable, and contact information for the study team and institutional review board for any questions, concerns, or requests for additional information. Study‐related examinations and treatments were provided at no cost to participants, and reasonable travel and accommodation expenses were reimbursed in accordance with institutional policy and ethics approval. Participants and their families did not receive additional monetary compensation. Inclusion criteria: (1) Aged 3–10 years; (2) Pathogenic *NCOR1/2* or *MECP2* mutations identified via whole‐exome sequencing (WES); (3) Willingness of participants/guardians to provide consent for clinical data collection, blood/urine sampling, and protocol adherence. Exclusion criteria: (1) Co‐occurring genetic variants with higher pathogenicity confidence than *NCOR1/2* or *MECP2* mutations; (2) History of spironolactone allergy or use within 1 month before enrollment; (3) Hyperkalemia (serum potassium >5.5 mmol/L) or renal dysfunction (eGFR <60 mL/min/1.73m^2^); (4) Active fever (body temperature >37.3°C) at screening; (5) Recent use (within 1 month) of medications affecting potassium homeostasis or spironolactone metabolism: potassium supplements, ACE inhibitors, angiotensin receptor blockers, digoxin, cholestyramine, acetylsalicylic acid, or abiraterone; (6) Clinically significant comorbidities (e.g., metabolic, hepatic, immunological, or neurological disorders) that could confound ASD‐related outcomes or compromise safety. Participants received oral spironolactone at a weight‐adjusted dose (2 mg/kg/day, maximum 50 mg/day). Serum potassium, renal function, and adverse events were monitored biweekly during the first month and monthly thereafter. Dose adjustments or discontinuation were implemented for potassium levels >5.0 mmol/L or intolerable side effects.

The cognitive and behavioral profiles of participants were evaluated using standardized neuropsychological tools administered by trained professionals or completed by parents/guardians. First, the core autism symptoms were assessed using three well‐validated instruments. The Autism Diagnostic Observation Schedule, Second Edition (ADOS2) [78], considered a gold‐standard diagnostic tool, is a structured observational assessment that evaluates key domains, including communication, reciprocal social interaction, play, and stereotyped behaviors or restricted interests. Higher scores reflect greater symptom severity. The Childhood Autism Rating Scale (CARS) [79] provides a clinician‐completed global evaluation of autism severity; total scores categorize individuals as non‐autistic (<30), mildly‐to‐moderately autistic (30–36.5), or severely autistic (≥37).

The Autism Behavior Checklist (ABC) [80] was administered to screen for autism spectrum traits. Parents rated 57 items across five domains: Sensory, Relating, Body and Object Use, Language, and Social/Self‐Help Skills. Total scores were classified to indicate the severity of autistic features. Adaptive functioning was evaluated using the Vineland Adaptive Behavior Scales, Third Edition (Vineland‐3) [81], which assesses Motor Skills (MOT), Communication (COM), Daily Living Skills (DLS), and Socialization (SOC). Parent‐reported scores were stratified into Very Superior (130–140), Superior (115–129), Average (86–114), Low (71–85), or Very Low (≤70). Language development was analyzed using the Chinese Communicative Development Inventory (CDI) [82], a parent‐reported questionnaire that quantifies expressive/receptive vocabulary, syntactic complexity, and gestural communication across multiple subdomains (e.g., verbs, spatial terms, phrases).

Behavioral challenges were assessed using the Conners Parent Symptom Questionnaire (CPRS) [83], which evaluates impulsivity‐hyperactivity, conduct problems, learning difficulties, psychosomatic symptoms, and anxiety. Total scores were categorized as Normal, Borderline, or Abnormal. The School‐Age Children's Behavioral Rating Scale of Executive Function evaluated multiple cognitive control processes, including Initiation (ability to begin tasks independently), Working Memory (capacity to hold and manipulate information), Inhibition (control over impulsive responses), and Organization (skills in structuring tasks and materials). Higher scores on this scale indicate better executive function performance in daily activities. All assessments adhered to standardized protocols, and data were analyzed using age‐adjusted normative references to ensure validity.

The treated patient underwent rigorous safety monitoring, including biweekly assessments of complete blood count (CBC), liver/renal function, serum electrolytes, blood pressure, and electrocardiography (ECG) during the first treatment month, followed by monthly evaluations thereafter. No adverse events (AEs) were observed throughout the 3‐month intervention. All biochemical parameters (e.g., ALT, AST, creatinine, potassium), hematologic indices, blood pressure, and ECG readings remained within age‐appropriate reference ranges (Table 2). Notably, serum potassium levels consistently ranged from 3.8 to 4.2 mmol/L despite chronic spironolactone administration, indicating effective renal homeostasis.

### 4.6 Statistical Analysis

Data analysis was performed using SPSS (v.21.0, IBM). For statistical significance, two‐tailed unpaired *t*‐tests or one‐way repeated ANOVA were used for experiments with two groups. Experiments with more than two groups were tested by one‐way ANOVA, two‐way ANOVA, or two‐way repeated ANOVA followed by Tukey's post hoc or Fisher's LSD post hoc tests for multiple comparisons. All experiments were performed at least twice using independent biological samples or distinct cohorts of mice, except the snRNA‐seq study, which was performed once. No statistical methods were used to pre‐determine sample sizes, but our sample sizes are similar to those reported in previous publications. All data met the assumptions of the statistical tests used. All data were tested for normality to determine the usage of parametric or nonparametric tests. Under the condition of nonequivalent variances, the nonparametric equivalent of the independent *t*‐test or nonparametric ANOVA was used to test the significance. Experimenters were not completely blinded to experimental conditions. The behavior test experimenter was blind to the genotype information, but not to the pharmacological agent. The statistical analysis and data plotting were then carried out by experimenters who knew both genotype and treatment information. All data were individually plotted (Prism 10, GraphPad). The exact N (number of animals) was described in the figure legend. *P *< 0.05 was considered to be significant. All data were expressed as means ± s.e.m. Animals were excluded and euthanized before behavior tests if they showed distress, infection, or injury due to fighting.

## Author Contributions

Y.K. and Z.S. conceived the study. Y.K. generated the NCOR1^KI/+^ mouse line, performed snRNA‐seq, and cell culture studies. W.Z. performed behavioral phenotyping analysis, gene expression analysis, and pharmacological rescue experiments. Y.S. and U.K.S. performed the snRNA‐seq data analysis. Z.H. performed the in vitro cell experiments. Z.L. executed the clinical studies and data analysis. C.L. and F.L. recruited patients. Y.L. performed the initial phenotyping of NCOR1^+/−^ mice. Z.H. consulted on snRNA‐seq analysis. Y.K., W.Z., and Y.S. analyzed data and performed statistical analysis. C.S., V.C.C., M.M., and L.E.K. assisted in mouse behavioral tests and mouse husbandry. Y.K., W.Z., Y.S., Z.L., A.C., and Z.S. interpreted the data. Z.S. obtained funding. Y.K., W.Z., Y.S., Z.L., A.C., and Z.S. wrote the manuscript with input from other authors.

## Conflicts of Interest

The authors declare no conflicts of interest.

## Supporting information




**Supporting File**: advs78030‐sup‐0001‐SuppMat.pdf.

## Data Availability

The data that support the findings of this study are available from the corresponding author upon reasonable request.
