# Supplementary material for: Drug Repurposing for Treating NCOR‐Related Intellectual Disability
Source: Adv Sci (Weinh). 2026 Oct 11:e78030. Online ahead of print. doi: 10.1002/advs.78030 (PMC13655318; doi:10.1002/advs.78030)
Supplement: Supplementary file 1 [file ADVS-9999-e78030-s001.pdf]

## Supplementary Figure 1

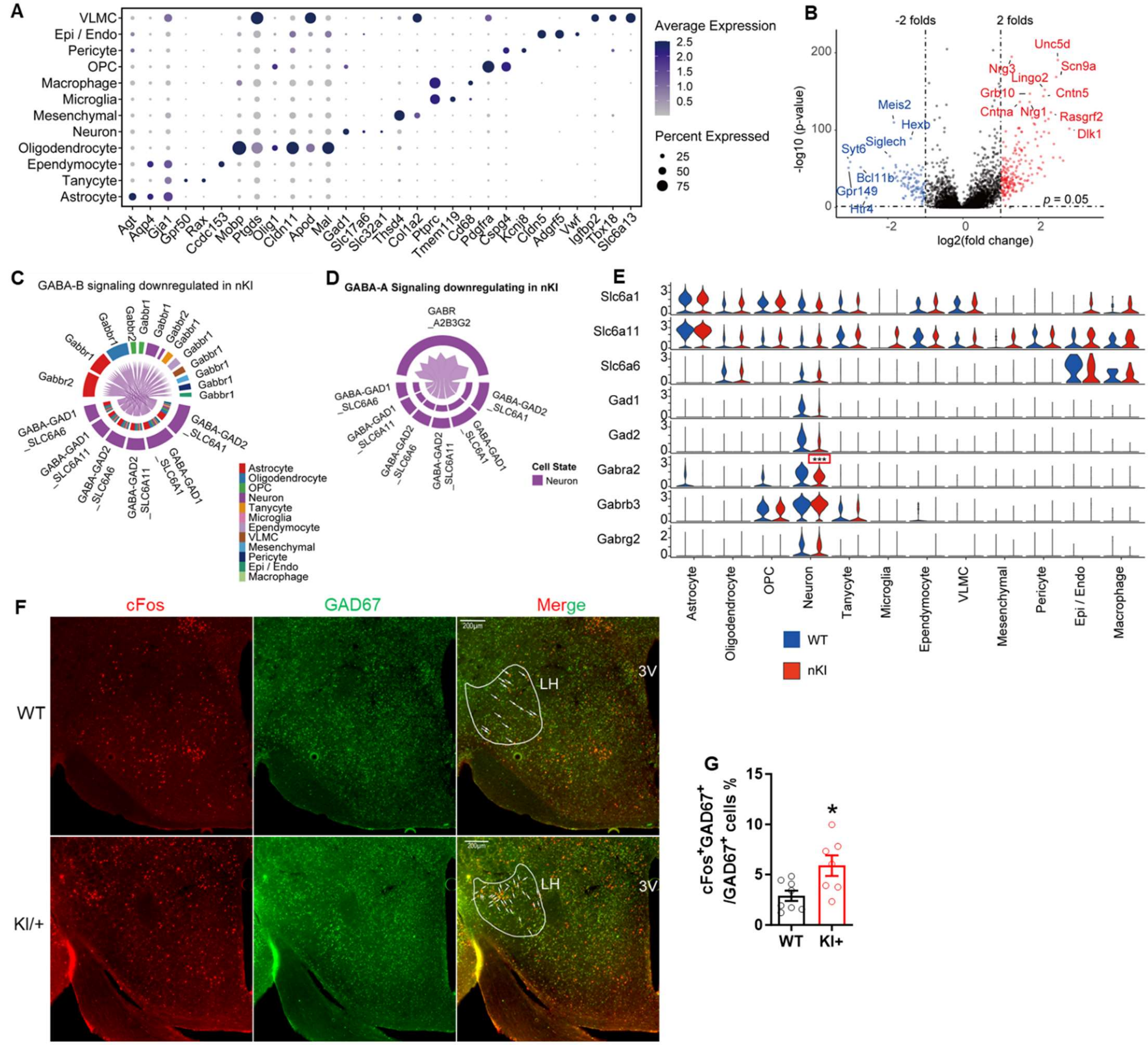


**Supplementary Figure 1**

**(A)** Canonical markers for cell clustering. **(B)** Differentially expressed genes in hypothalamic neurons of WT and NCOR1$^{KI/+}$ mice. **(C-D)** The chord plots show details of ligand-receptor pairs in hypothalamic neurons from knock-in mice compared to WT, with downregulated GABA-B signaling and GABA-A signaling. **(E)** Expression levels of genes in GABA-A signaling in different hypothalamic cell types of WT and NCOR1$^{KI/+}$ mice. **(F-G)** Immunostaining and quantification of cFos and GABAergic neuron marker GAD67 in the lateral hypothalamus (LH) of WT and KI/+ mice (n = 7 mice). 3V, third ventricle. Arrows indicate the cFos$^{+}$/CAD67$^{+}$ cells in the LH. **P*<0.05 by a two-tailed unpaired t-test.

## Supplementary Figure 2

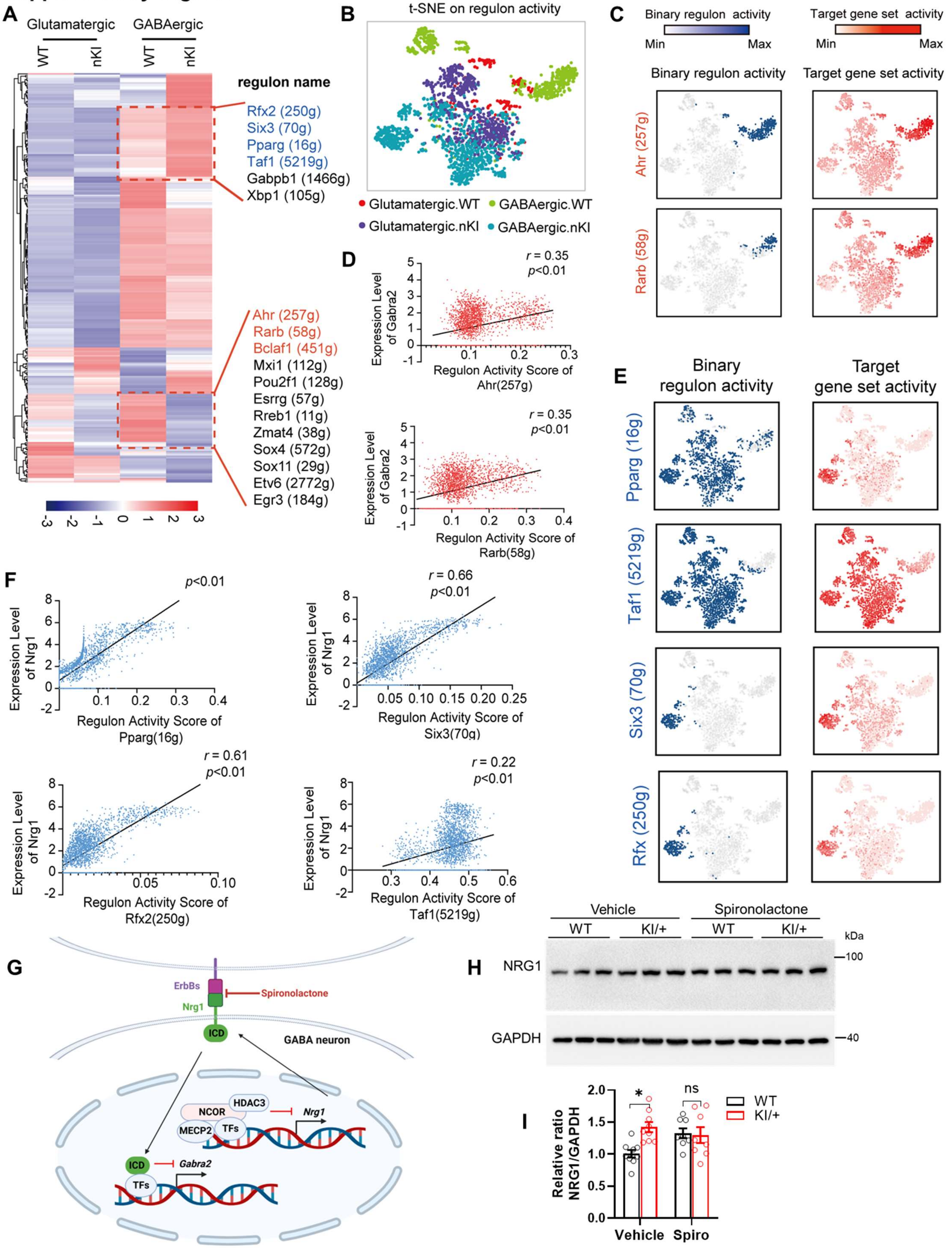

**Supplementary Figure 2. (A)** The heatmap shows the activity of regulons in GABAergic and glutamatergic neurons of WT and NCOR1$^{KI/+}$ mice. Regulons were annotated by TFs. Genes in the regulons targeting Nrg1 are highlighted in blue, and genes targeting *Gabra2* are highlighted in red. **(B)** t-SNE plot on binary regulon activity matrix of neuron sub-clusters. Each nucleus is colored according to the corresponding subtype. **(C)** t-SNE on the binary regulon activity matrix (left panel) and target gene set activity (right panel) of select regulons in neurons. **(D)** The scatter plots show correlations between selected TF activities and *Gabra2* expression levels in GABAergic neurons. **(E)** t-SNE on the binary regulon activity matrix (left panel) and target gene set activity (right panel) of select regulons in neurons. **(F)** The scatter plots show correlations between selected TF activities and *Nrg1* expression levels in GABAergic neurons. (**G**) Working model. The NCOR complex is recruited by TFs such as *Pparg*, *Taf1*, *Six3*, and *Rfx*. NCOR deficiency upregulates Nrg1, which downregulates *Gabra2* expression through functional interplay between NRG1-ICD and other TFs such as *Ahr* or *Rarb*. **(H-I)** Western blot and quantification of NRG1 protein level in the brain of WT and NCOR1$^{KI/+}$ mice. n = 7-8 mice. *$P$<0.05 by two-way ANOVA and Sidak post hoc test.
